# Thirst Is Associated with Suppression of Habenula Output and Active Stress Coping: Is there a Role for a Non-canonical Vasopressin-Glutamate Pathway?

**DOI:** 10.3389/fncir.2016.00013

**Published:** 2016-03-31

**Authors:** Limei Zhang, Vito S. Hernández, Erika Vázquez-Juárez, Freya K. Chay, Rafael A. Barrio

**Affiliations:** ^1^Departamento de Fisiología, Facultad de Medicina, Universidad Nacional Autónoma de MéxicoCiudad de México, Mexico; ^2^Departamento de Física Química, Instituto de Física, Universidad Nacional Autónoma de MéxicoCiudad de México, Mexico

**Keywords:** interneuron, fluorogold, juxtacellular labeling, homeostasis, motivation, dense core vesicle, synapsis, metastress coping

## Abstract

Water-homeostasis is a fundamental physiological process for terrestrial life. In vertebrates, thirst drives water intake, but the neuronal circuits that connect the physiology of water regulation with emotional context are poorly understood. Vasopressin (VP) is a prominent messenger in this circuit, as well as L-glutamate. We have investigated the role of a VP circuit and interaction between thirst and motivational behaviors evoked by life-threatening stimuli in rats. We demonstrate a direct pathway from hypothalamic paraventricular VP-expressing, glutamatergic magnocellular neurons to the medial division of lateral habenula (LHbM), a region containing GABAergic neurons. *In vivo* recording and juxtacellular labeling revealed that GABAergic neurons in the LHbM had locally branching axons, and received VP-positive axon terminal contacts on their dendrites. Water deprivation significantly reduced freezing and immobility behaviors evoked by innate fear and behavioral despair, respectively, accompanied by decreased Fos expression in the lateral habenula. Our results reveal a novel VP-expressing hypothalamus to the LHbM circuit that is likely to evoke GABA-mediated inhibition in the LHbM, which promotes escape behavior during stress coping.

## Introduction

It is commonly observed that animals experiencing severe physiological stress, such as prolonged food and water deprivation, are prepared to take greater risks to fight or take flight for survival, whereas in a satiated state, passive stress coping strategies generally predominate. This phenomenon suggests that internal homeostatic demands may exert powerful modulation on emotion, motivation, and motor circuits.

Thirst is a fundamental function of the nervous system aiming to maintain the internal water, salt and blood pressure homeostasis. Thirst is regulated by many factors that convey to the metabolic activation of the hypothalamic magnocellular neurosecretory neurons (MNNs) in the paraventricular (PVN) and supraoptic (SON) nuclei (McKinley and Johnson, 2004; McKinley et al., 2004). The activated MNNs release the nonapeptide arginine vasopressin (VP) to the vascular plexus of the neurohypophysis exerting peripheral endocrine functions such as the increase of water re-absorption in the kidney and contraction of smooth muscles of blood vessels to enhance the blood pressure (Armstrong, 2004). Aside the neuroendocrine functions, VP-MNNs' intracerebral micro-circuitry is not fully understood.

It is widely accepted that the lateral habenula (LHb) codifies negative motivational value and its over-activation fosters depression-related behavior (Matsumoto and Hikosaka, 2007; Hikosaka, 2010; Li et al., 2011; Lecca et al., 2014). The main afferent and efferent connections of the mammalian LHb comprise the so called “dorsal diencephalic conduction system” (Sutherland, 1982), via which the forebrain structures, most importantly the forebrain and midbrain limbic regions influence the neuronal activities in the midbrain aminergic regions (Hikosaka et al., 2008). Most LHb afferents arrive via the *stria medullaris* (sm) from basal ganglia, entopeduncular nucleus, nucleus accumbens, preoptic regions of the hypothalamus, and septum (Herkenham and Nauta, 1977). Virtually all efferents project via the *fasciculus retroflexus* (fr) to various midbrain structures including the dorsal raphe, the *substantia nigra pars compacta*, and the ventral tegmental area (Herkenham and Nauta, 1979). Electrical stimulation of the LHb has a marked suppressant effect on the spontaneous activity of most serotonergic and dopaminergic neurons in the midbrain (Wang and Aghajanian, 1977), suggesting that an important function of the LHb is the modulation of the monoaminergic neuronal activity.

The presence of VP containing axons and terminals in the LHb has long been observed (Buijs, 1978). However, the origin of these projections, which neurons they act on, and the functional implications of these connections are still unclear. Here, by using anatomical and immunohistochemical methods, we found that the medial subdivision of lateral habenula (LHbM) was a main VP fiber-targeting region, which concurred with a unique population of GABAergic interneurons in the habenular complex. At the electron microscopy level, VP+ terminals were found establishing Gray type I synapses with VP containing vesicles docked on the presynaptic membrane suggesting a role for synaptic transmission. With *in vivo* juxtacellular labeling and reconstruction methods, we observed that GABA containing neurons located in the LHbM possessed axons that branched extensively in the LHbM parvocellular subnucleus (LHbMPc), and that their dendrites received VP+ fiber contacts. Fluorogold retrograde tracing from LHbM showed that the VP-MNNs of PVN served as one of the sources for VP habenular innervation. This notion is further confirmed by *in vivo* juxtacellularily labeled and reconstructed VP-MNNs. We therefore, hypothesized that modulation of thirst would significantly influence the information processing concerning innate fear and behavioral despair through activation of a habenular microcircuit,. Indeed, recent studies have reported that hunger promotes fear extinction through activation of an amygdala microcircuit (Verma et al., 2016) and that hypernatremia attenuates the cardiovascular response to restraint and promotes the recovery to pre-stress levels (Krause et al., 2011). Our results shows that up-regulation of the metabolic activity of the VP-MNNs by 24 h water-deprivation (WD24) reduced freezing/immobility behaviors during live predator (cat) exposure and forced swimming test (FST), which correlated with reduced Fos expression in the whole lateral habenular complex, suggesting a down-regulation of LHb's functional output in response to WD24.

## Materials and methods

### Animals

Adult male Wistar rats of 280 g ± 20 g (except for the *in vitro* patch clamp experiments, in which 3 weeks old rats were used), were obtained from the local animal facility. Animals were housed three per cage under controlled temperature and illumination (12 h/12 h, light/dark cycle) with water and food *ad libitum*. All animal procedures were approved by the local bioethical and research committees (CIEFM-086-2013).

### Immunohistochemistry

Rats were deeply anaesthetized with an overdose of sodium pentobarbital (63 mg/kg, Sedalpharma, México) and perfused transaortically with 0.9% saline followed by cold fixative containing 4% of paraformaldehyde in 0.1 M sodium phosphate buffer (PB, pH 7.4) plus 15% v/v saturated picric acid for 15 min (for immunoreaction using monoclonal antibody against GABA, the fixative was added 0.1% of glutaraldehyde additionally). Brains were immediately removed, blocked, and then thoroughly rinsed with PB. Brains were sectioned soon after perfusion using a Leica VT 1000S vibratome. Freshly-cut freely-floating sections were blocked with 20% normal donkey serum (NDS) in Tris-buffered (0.05 M, pH 7.4) saline (0.9%) plus 0.3% of Triton X-100 (TBST) for 1 h at room temperature and incubated with the primary antibodies listed in Table 1 (for antibody specificity see supplementary information Table S1). For light microscopy immunohistochemistry, Vectastain Elite ABC Kit (Vector Labs, Burlingame, CA) followed by DAB-peroxidase reaction was done while for immunofluorescence reactions, sections were incubated with the corresponding fluorochrome-conjugated secondary antibodies. For immunoreaction imaging analysis, Nikon Eclipse 50i light microscope and Leica TCS-SP5 confocal microscope were used.

**Table 1:** **Antibody information, related to Materials and Methods**.

**Molecule**	**Host species**	**Dilution**	**Source**	**Source code**
[Arg8]-vasopressin	Rabbit	1:5000	Peninsula-Bachem Americas, Inc., CA, USA. (http://www.bachem.com)	T-4563
[Arg8]-vasopressin	Rabbit	1:2000	Prof. R.M. Buijs, Mexico City, Mexico	–
Tyrosine Hydroxylase	Mouse	1:2000	Sigma-Aldrich Corporation, MO, USA	T-2928
Dopamine β-hydroxylase (DβH	Rabbit	1:1000	EMD Millipore Corporation, Billerica, MA, USA (http://www.millipore.com)	AB-1585
Serotonin Transporter (SerT)	Goat	1:2000	Santa Cruz Biotechnology, Dallas, Texas U.S.A (http://www.scbt.com)	SC-1458
Somatostatin (Som)	Mouse	1:1000	GeneTex, Inc., CA, USA (http://www.genetex.com/)	GTX7-1935, clone SOM-018
Enkephalin (Enk)	Mouse	1:1000	EMD Millipore Corporation, Billerica, MA, USA (http://www.millipore.com)	MAB-350
Substance P (SP)	Rat	1:1000	EMD Millipore Corporation, Billerica, MA, USA (http://www.millipore.com)	MAB-356
Calretinin (CR)	Goat	1:1000	Swant Swiss antibodies, Marly, Switzerland (http://www.swant.com)	CG1
Calbindin	Rabbit	1:5000	Swant Swiss antibodies, Marly, Switzerland (http://www.swant.com)	CB38
G-protein regulated Inward-Rectifier K+ channel, (GIRK1)	Rabbit	1:1000	Alomone Labs, Jerusalem, Israel (http://www.alomone.com)	APC-005
G-protein regulated Inward-Rectifier K+ channel, (GIRK2)	Rabbit	1:1000	Alomone Labs, Jerusalem, Israel (http://www.alomone.com)	APC-006
c-Fos	Rabbit	1:2000	Santa Cruz Biotechnology, Dallas, Texas U.S.A (http://www.scbt.com)	SC-52
Vesicular glutamate transporter 2	Guinea pig	1:1000	Frontier Institute Co., Ltd., Hokkaido, Japan (http://www.frontier-institute.com)	GP-AF240-1
Glutamate receptor 1C (GluR1c)	Rabbit	1:1000	Frontier Institute Co., Ltd., Hokkaido, Japan (http://www.frontier-institute.com)	GluR1C-Rb-Af692
Gamma-aminobutyric Acid (GABA)	Mouse	1:1000	EMD Millipore Corporation, Billerica, MA, USA (http://www.millipore.com)	MAB-316
Gamma-aminobutyric Acid (GABA)	Mouse	1:1000	Sigma-Aldrich Corporation, MO, USA (http://www.sigmaaldrich.com)	A0310
Glutamic acid decarboxylase 65 kDa isoform (GAD 65)	Mouse	1:2000	EMD Millipore Corporation, Billerica, MA, USA (http://www.millipore.com)	MAB351
Glutamic acid decarboxylase 65 kDa isoform (GAD 67)	Mouse	1:2000	EMD Millipore Corporation, Billerica, MA, USA (http://www.millipore.com)	MAB5406

### Neuronal activation through fos-expression assessment

The pattern of habenular neuronal activation was assessed by measuring the Fos+ nuclei in the whole LHb, sampled at three rostro-caudal levels, namely, rostral (around Bregma − 3.14 mm), middle (around Bregma −3.60 mm) and caudal (around Bregma −3.96 mm) of 6 experimental groups: basal (*n* = 4), WD24 (*n* = 4), cat exposure (*n* = 8), cat exposure + WD24 (*n* = 8), FST (*n* = 5), FST + WD24 (*n* = 7). A *t*-test was used to evaluate if the differences between basal vs. WD24, cat exposure vs. cat exposure+WD24 and FST vs. FST+WD24 were statistically significant.

### Immunonohistochemistry for transmission electron microscopy (TEM)

Immunoelectron microscopy procedures were performed as reported previously (Zhang and Hernández, 2013). Briefly, rats were deeply anesthetized with an overdose of sodium pentobarbital (63 mg/kg, Sedalpharma, México) and then perfused first with 0.9% saline, followed by a fixative containing 4% paraformaldehyde, 15% v/v saturated picric acid and 0.05% glutaraldehyde in 0.1 M sodium phosphate buffer (PB, pH 7.4) for 15 min. Coronal sections 70 μm thick containing lateral habenula were selected. The non-specific antibody binding was blocked with 20% normal swine serum (NSS) in TBS + Triton-X-100 0.025% for 1 h. The sections were then incubated with a cocktail of two rabbit anti-arginine vasopressin (AVP) antibodies (from Prof. R. M. Buijs, 1:2000 and from Peninsula Laboratories T-4563, 1:5000) in TBS plus 1% NSS for 48 h at 4°C with gentle shaking. Sections were then rinsed and proceeded to the secondary antibody incubation with swine anti-rabbit IgG conjugated with horseradish peroxidase (HRP) (P021702, 1:100, Dako, Denmark) in TBS containing 1% NSS, overnight at 4°C. Peroxidase enzyme reaction was carried out using the chromogen 3, 3′–diaminobenzidine (DAB, 0.05%, Electron Microscopy Sciences, Hatfield, PA) and hydrogen peroxide (H2O2, 0.01%) as the substrate. Sections were then post-fixed with 1% osmium tetroxide in 0.1 M PB for 1 h and dehydrated through a series of graded alcohols (including 45 min of incubation in 1% uranyl acetate in 70% ethanol), then transferred to propylene oxide, followed by Durcupan ACM epoxy resin (Electron Microscopy Sciences). Sections were flat embedded on glass microscope slides, and the resin was polymerized at 60°C for 2 days. Areas containing AVP immunolabeled axons in the medial part of the lateral habenula were re-embedded in capsules with Durcupan resin. Ultrathin sections (~70 nm) were cut with an ultramicrotome using a diamond knife. Sections were collected onto Pioloform-coated single slot grids and examined with a Philips CM100 transmission electron microscope. Digital electron micrographs were obtained with a digital micrograph 3.4 camera (Gatan, Inc., Pleasanton, CA) and scaled with ImageJ (Image Processing and analysis in Java, Bethesda, NIH, USA) and Adobe Photoshop.

### Fluoro-gold tracing experiments

Anesthesia was induced and mantained with xylazine (Procin, Mexico; 20 mg/ml) and ketamine (Inoketam, Virbac, Mexico; 100 mg/ml) mixed in a 1:1 volume ratio and administered intramuscularly with a dose of 1 ml/kg body weight. Rats were fixed in a stereotaxic apparatus and were injected in the LHbMC [Bregma −3.50 mm, medio-lateral 0.50 mm, dorso-ventral 4.30 mm (Paxinos and Watson, 2006)] with the retrograde tracer Fluoro-Gold (FG, Fluorochrome, LLC, Denver, Colorado 80218 USA), dissolved 1% in 0.2 M sodium cacodylate buffer (pH 7.5). The FG was delivered iontophoretically using an iontophoresis pump (Value Kation Sci VAB-500) through a stereotaxically positioned glass micropipette with an inner tip diameter of about 40 μm, by applying current pulses of 0.1 μA, at 0.2 Hz, with a 50% duty cycle for 20 min. The micropipette was left in place for an additional 10 min to prevent backflow of the tracer up the injection track after each injection. After completing the surgery, rats received 0.4 mg/kg i.p. ketorolac (Apotex, Mexico) and 50 mg/kg i.p ceftriaxone (Kendric, Mexico) as analgesic/anti-inflammatory and antibiotic agents. Three to four weeks after the FG injections, the rats were perfused as previously described (Zhang and Hernández, 2013). Coronal and sagittal sections of 70 μm were obtained, and VP IHC was performed to evaluate if the SON and PVN VP+ neurons were labeled with FG. Observations were made under light (Nikon ECLIPSE 50i with B-2A long-pass emission filter) and confocal microscopy (LSM 710, DUO, Carl Zeiss, from Instituto Nacional de Cancerología, Mexico). For confocal observation the FG at the target regions was excited with a 452 nm Argon filter (Zeiss).

### Colchicine intra-habenular injection

Colchicine has been used to locally increase the concentration of GAD in neuronal somata (Ribak et al., 1978). The effectiveness of this procedure relies in the property of colchicine to interrupt axonal transport. For this study, 4 rats were deeply anesthetized with a 1.5 g/kg dose of urethane *i.p.* (25% in NaCl 0.9%), and secured in a stereotaxic apparatus, local anesthesia with lidocaine was performed before making a craniotomy around the coordinates Bregma –3.5 mm, lateral 0.5 mm and dorsoventral 4.3 mm. The pipette was lowered with a micro positioner (2660, Kopf) until reaching the medial part of lateral habenula's coordinates. Colchicine (1 μg/1 μl in saline 0.9%, Sigma-Aldrich, C9754) was injected at a rate of 1 μl/min during 10 min through a glass pipette with a tip diameter of 30–50 μm connected to a syringe pump (WPI SP101i), after the completion of the injection, 10 min were allowed before withdrawing the pipette, to avoid back flow. Rats were kept at 35°C with a temperature controller (TCAT-2LV, Kopf), for 6 h until transcardial perfusion/fixation procedures.

### RNAscope ISH assays

Two rats were deeply anesthetized and transcardially perfused with saline. Whole brain tissues were removed and rapidly frozen on dry ice. The fresh-frozen tissue sections (20 μm thick) were mounted on positively charged microscopic glass slides (Fisher Scientific, Pittsburgh, PA). Both the vasopressin V1a and Gad1 specific RNA probes (Rn-Avpr1a, 402531 and Rn-Gad1, 316401) were designed and provided by Advanced Cell Diagnostics (Hayward, CA). All staining steps were performed following RNAscope protocols. Stained slides were coverslipped with fluorescent mounting medium (ProLong Gold Antifade Reagent P36930; Life Technologies) and examined with a confocal microscope (Leica TCS-SP5) at 63x magnification using the manufacturer-provided software.

### *In vivo* juxtacellular labeling of LHbMC neurons

The procedures for *in vivo* juxtacellular labeling were based on the methods described in references (Leng and Dyball, 1991; Pinault, 1996; Tukker et al., 2013). Anesthesia was induced with 4% isoflurane in oxygen, followed by urethane injection (1.3 g/kg, *i.p.*, Sigma-Aldrich), with supplemental doses of xylazine (30 mg/kg) as necessary. Body temperature was maintained at 36°C with a heating pad. Once anesthetized, animals were placed into a stereotaxic apparatus and craniotomy was performed around the coordinates: −3.5 mm posterior from Bregma and 0.5 mm lateral. A long-taper glass electrode (8–15 MΩ) filled with 1% neurobiotin (Vector Laboratories), in 0.15 M NaCl was vertically placed at previously standardized hypothalamic LHb medial coordinates (3.6 mm posterior to Bregma, 0.5 mm right/left from midline and 4.3 mm deep) and referenced against a wire implanted subcutaneously in the neck.

Neuronal activity was detected, amplified and filtered for single unit recording using differential amplifiers ELC-01MX and DPA-2FL (NPI electronics, GmbH, Tamm, Germany). When a neuron was successfully isolated, it was iontophoretically labeled with neurobiotin using the juxtacellular-labeling method (Pinault, 1994). Current pulses of 1–10 nA, at 2.5 Hz, with a 50% duty cycle, were delivered through the recording electrode. The current was gradually increased to induce and maintain entrainment of the activity of the neuron, yielding a higher number of spikes on the current “*on*” periods. Cells were entrained between 2 and 10 min. After the procedure, the rats were maintained at 35°C during 4–6 h before perfussion, to allow an extensive diffusion of the Neurobiotin through the neurites.

### *In vitro* whole cell current-clamp recording

Brain slices were prepared from male 21-days old Wistar rats. Animals were deeply anesthetized with sevoflurane (SevoFlo, Abbott Las, Abbott Park, Illinois) and decapitated. Brains were quickly dissected and sectioned coronally at 300 μm with a vibrating microtome (Leica VT1000 Leica Instruments, Nussloch, Germany), the solutions used for these steps were based on Weiss and Veh (2011). The cutting solution contained (in mM): 75 NaCl, 50 Sucrose, 25 NaHCO3, 2.5 KCl, 1.25 NaH2PO4, 0.1 CaCl2, 6 MgCl2, and 25 D-glucose, pH 7.4. The recording solution contained (in mM): 125 NaCl, 25 NaHCO3, 2.5 KCl, 1.25 NaH2PO4, 2 CaCl2, 2 MgCl2, and 25 D-glucose, pH 7.4. The recording was performed at 35°C.

Patch-clamp recordings were made from individual neurons visualized by differential interference contrast video microscopy using a CCD camera (CCD-100; DAGE-MTI, Michigan, IN) mounted to an upright microscope (Nikon Eclipse E-600 FN) with a 40x water immersion objective (Nikon), and an infrared filter (Nikon). Patch pipettes were made from borosilicate glass tubing (outer diameter, 1.5 mm; inner diameter 0.86 mm; Sutter Instruments, BF150-86-10HP, Novato, CA) using a Sutter micropipette puller (P-97; Sutter Instruments, Novato, CA, USA). When filled with internal solution (128 K-gluconate, 20 KCl, 10 HEPES, 0.1 EGTA, 2 MgCl2, and 2 Na2ATP, 2 mg/ml neurobiotin, and pH adjusted to 7.2 with NaOH) pipettes had resistances of 5–8 MΩ. Whole cell recordings were performed employing an Axoclamp 2B amplifier (Molecular Devices, Sunnyvale, California). Data acquisition was controlled with Clampex (PCLAMP10, Molecular Devices). Signals were digitized at 20 kHz.

The parameters investigated in this study were: resting membrane potential (*V*m), membrane input resistance (*R*m), and discharge response upon depolarizing stimulation. Once a stable recording was obtained, the series resistance was monitored throughout the experiment. If the series resistance of the electrode was unstable or exceeded four times the electrode resistance, electrophysiological data from the cell were discarded. Only one cell per slice was recorded. Baseline input resistance was derived from the linear portion of a voltage-current plot of hyperpolarizing current steps. Discharge response upon depolarizing stimulation was analyzed in response to a protocol that consisted in a series of 250 ms pulses of current injection from −50 to +60 pA, in 10 pA increments. The interval between stimuli was 10 s. For firing frequency measurement, spikes with overshoot beyond 0 mV were counted as action potentials. The protocol was repeated 3 times with the neuron under ACSF perfusion, 8 min after the administration of VP 10 nM (American Peptides Company, Vista, CA), and after a 10 min wash of the peptide.

Data were analyzed with *Clampfit* software (Molecular Devices, Sunnyvale CA). All data are reported as mean ± standard error of the mean (SEM). To evaluate statistical significance, data were subjected to Student's *t*-test. A probability value of *P* < 0.05 was considered to be significant.

### Live cat exposure and behavioral scoring

The behavior test was performed during the early activity period of the rats (dark period). Experimental subjects housed three per cage, were divided into two groups: the undisturbed control group and the 24 h water deprivation group (WD24). For innate fear assessment, each rat was placed individually in a grid cage (28.5 × 21 × 30 cm), so the rat could climb. The cage was placed inside a larger ventilated clear plastic chamber (60 × 80 × 40 cm), where a male adult cat was then introduced. The cat was tamed and castrated, about 5 kg of body weight. The cat was mostly kept quiet/immobile during the experiment. One advantage of this arrangement is that the rats were exposed to physiologically relevant stimuli—a live predator's odor, appearance and breathing sounds, which were relatively constant for all the experimental subjects. Each rat remained in the chamber described above for a single period of 10 min. Once the time of exposure was completed, they were returned to their home cage.

Relevant behaviors were quantified offline by giving one of the six scores every 5 s: (1) “Freezing” was assigned to the behavior of immobility for more than 2 s with pilo-erection; (2) “Climbing”: when rats climbed the internal mesh cage using limbs trying to escape from the top door; (3) “Rearing,” when rats were rearing still, sniffing with short head rotations; (4) “Displacement,” when the rats were walking, trotting or running; (5) “Orientation”: when the four limbs of the rats were still with head extension, rotation; (6) “Grooming”: when rats groomed themselves (nose, head, face, eyes, and body) with their paws, using very short movements. “Active Escaping” measured in the study included the behaviors 2–4.

### Forced swimming test

In this test, rats under basal and with 24 h of water deprivation (WD24) were placed into 45 cm height × 30 cm diameter Plexiglas cylinders, filled with water at 25°C, up to a height of 25 cm, and their behavior was recorded over a 6-min test period. The behavior was evaluated offline using the criteria described previously (Detke et al., 1995; Zhang et al., 2008). Briefly, the observers scored the behaviors as one of the 3 classes, “swimming,” “climbing,” and “immobility,” every 5 s. “Climbing” was scored as movements directed toward the top edge of the cylinder and “immobility” was considered when there was cessation of spatial displacement with or without minor involuntary movements of the hind limbs.

### Statistical analysis

Quantitative results were expressed as mean ± SEM. Groups were tested for normality with a D'Agostino and Pearson test, then differences between groups were calculated by Student *t*-test or analysis of variance followed by the Bonferroni test, using Prism (GraphPad Software, San Diego, CA, USA). Differences were considered statistically significant at (^*^P < 0.05, ^**^P < 0.01, and ^***^P < 0.001).

## Results

### VP+ fibers densely innervate the LHbM

**Figure 1:**
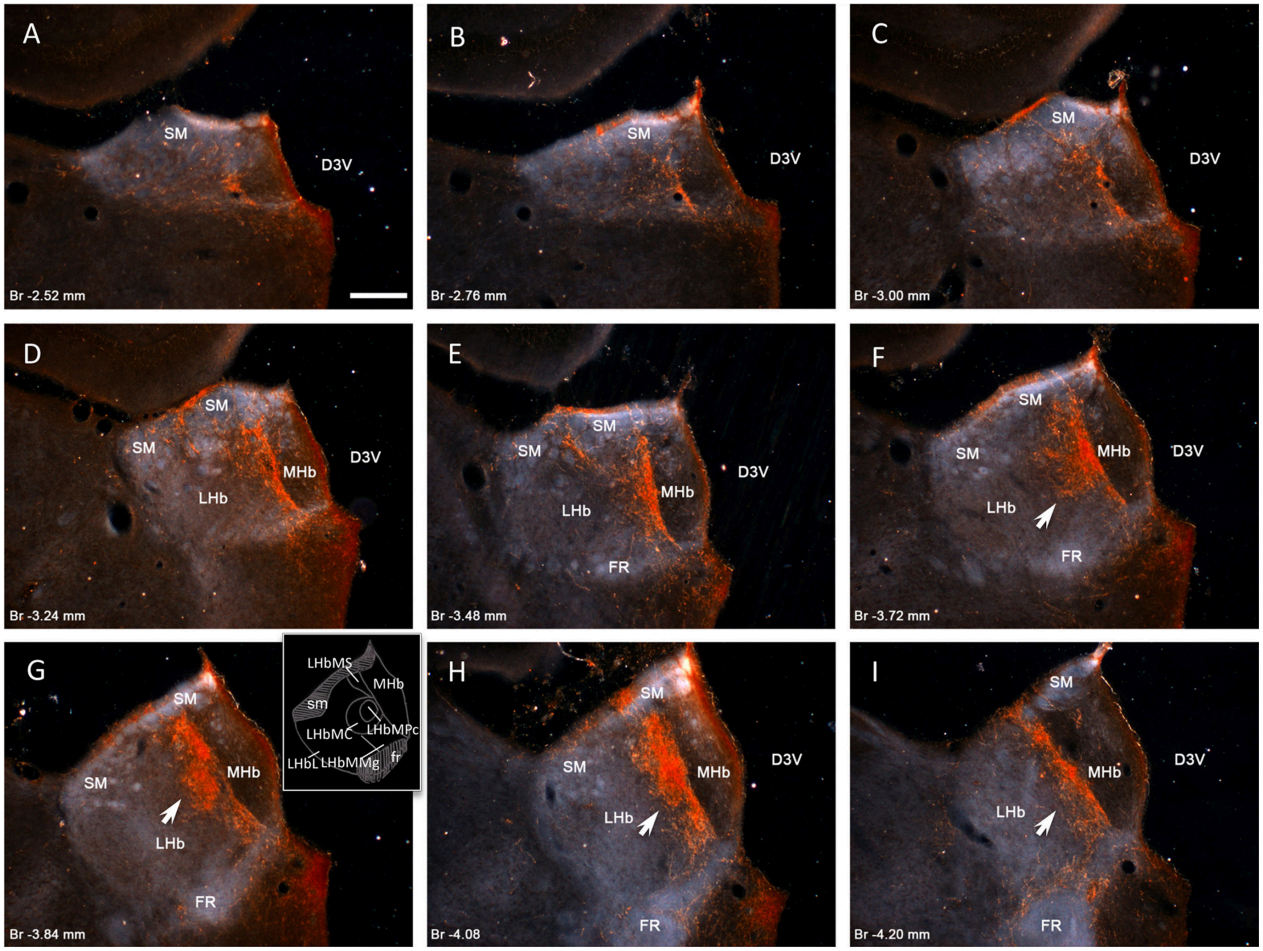
**Vasopressin immunopositive fiber distribution through the rostro-caudal extent of rat habenular complex**. Panels **(A–I)** dark-field photomicrographs of AVP immunoreaction on coronal sections at the rostro-caudal Bregma levels indicated in each panel. Vasopressin immunopositive fibers densely innervate the medial division of lateral habenular complex (LHbM), i.e., the superior subnucleus (LHbMS), the central subnucleus (LHbMC), remarkably its parvocellular subnucleus (LHbMPc), and the marginal subnucleus (LHbMMg). Inset of the panel **(G)** shows a drawing with the subnuclei mentioned. Note that highest density of AVP+ fibers is observed inside the LHbMC (panels **F–H** arrows). Scale bars: 250 μm. D3V, dorsal 3rd ventricle; MHb, medial habenular complex; LHb, lateral habenular complex; SM, stria medularis; FR, fasciculus retroflexus.

We used anti-VP antibodies to characterize the VP immunopositive fiber distribution and found a highly selective distribution pattern in the medial subdivision of the LHb (LHbM) (Figure 1). The fibers were grouped mainly in three subnuclei: the superior subnucleus (LHbMS), the central subnucleus (LHbMC), and the marginal subnucleus (LHbMMg) (see inset of panel G). Notice that the highest density of VP+ fibers was observed inside the LHbMC (Figures 1F–I, arrows). For the sake of completeness, we also examined the expression of other relevant neurochemical markers: tyrosine hydroxylase (TH), dopamine beta hydroxylase (DBH), serotonin transporter (SerT), somatostatin (SOM), enkephalin (ENK), substance P (SP), calretinin (CR), calbindin (CB), G protein-coupled inwardly-rectifying potassium channel 1 and 2 (GIRK1 and GIRK2) (see supplementary information Figure S1). Interestingly, a strong regional innervation overlapping pattern was observed within the LHbMC between VP and midbrain aminergic projections (immnoreactivity to TH and SerT, but not DBH which was sparse and not regional–specific) (supplementary information SI, Figures S1A–C). This phenomenon may indicate that: (1) the LHbMC is a key region modulated by subcortical aminergic pathway; (2) the VP innervation may contribute to the robustness of the modulatory mechanism for LHb function, which is also regulated by midbrain aminergic pathways. The region of strong immunostaining for SOM, CR, and CB was also similar to VP. The expressions of ENK, SP, and GIRK1 and GIRK2 were not similar to that of VP, particularly low in LHbMC. CB immunopositive somata were predominantly located in the LHbMC and CB+ axons seemed to project to fasciculus retroflexus.

### VP+ fibers co-expressed VGluT2 and established gray type I synapses onto habenular neurons' dendrites

**Figure 2:**
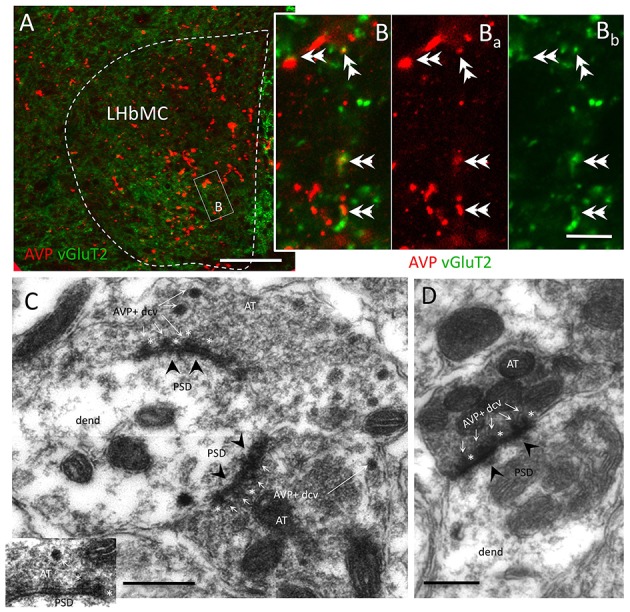
**Most AVP+ axon terminals co-expressed vesicular glutamate transporter 2 (vGluT2) and established Gray type I synapses onto habenular neuron's dendrites**. **(A,B)** Representative confocal photomicrographs of double immunofluorescence AVP (red) and vGluT2 (green) centered at the medio-central lateral habenular (LHbMC) subnucleus. Double arrowheads indicate the double-labeled axon terminals. **(C,D)** Electron microscopy photomicrographs showing the AVP+ dense core vesicles (dcv, thin white arrows) inside the axon terminals (AT) established Gray type I synapse (post-synaptic densities, PSD, were indicated with black arrowheads) onto habenular neuron's dendrites (dend). Asterisks are put adjacent to AVP+ dcv, which showed docking onto presynaptic membranes. Scale bars: **A**: 50 μm; **B**: 5 μm, **C**, **D**: 500 nm.

Vesicular glutamate transporter 2 (vGluT2 belongs to a family of three vesicular glutamate transporters (vGluT1, vGluT2, and vGluT3). The vGluT2 is the subtype expressed in the hypothalamic neuroendocrine magnocells and exhibit robust up-regulation in response to certain homeostatic challenges (Ziegler et al., 2002; Hrabovszky and Liposits, 2007, 2008). Using double immunofluorescence and confocal microscopy, we have observed that most of the VP+ axon terminals co-expressed vGluT2 (Figures 2A,B). At the level of electron microscopy, we found that VP containing axon terminals in the LHbMC region established Gray type I (asymmetric) synapses (10:10, n:N) onto dendrites of habenular neurons (Figures 2C,D). Interestingly, some VP+ dense core vesicles (dcv) were found in co-storage in the active zone, adjacent to the presynaptic membranes and some seemed to be docked onto the presynaptic membrane (Figures 2C,D asterisks).

### VP+ magnocells from SON and PVN innervated the LHbM

**Figure 3:**
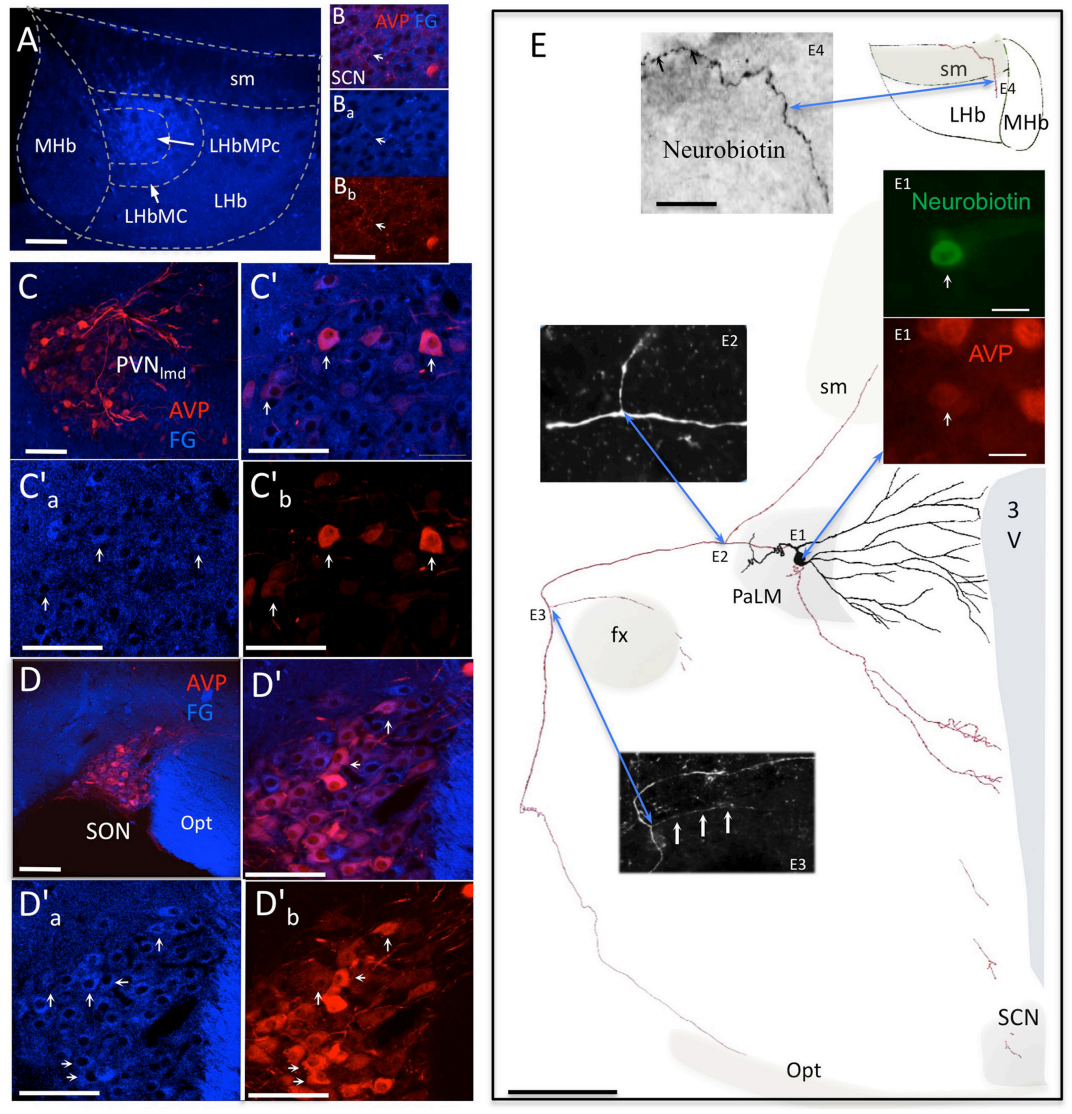
**AVP containing magnocellular neurosecretory neurons serving as one of the sources of AVP+ axons in LHb**. **(A)** Fluoro-Gold (FG) retrograde tracer was injected in the medio-central subnucleus of the lateral habenula (LHbMC). **(B)** In the hypothalamic suprachiasmatic nucleus (SCN, panels **B′a–b**), only sparse double-labeled cellular components were found. **(C)** Numerous FG+/AVP+ somata were found in hypothalamic paraventricular nucleus (PVN, **(C′)** shows an magnification of the region and (**C**${}_{\text{a}}^{\prime }$**)** and **(C**${}_{\text{b}}^{\prime }$**)** are the separated channels). Arrows indicate some double-labeled cells. **(D)**, **(D′)**, **(D**${}_{\text{a}}^{\prime }$**)**, and **(D**${}_{\text{b}}^{\prime }$**)**. Same cases of Cs but in the hypothalamic supraoptic nucleus (SON). (**E)** camera lucida reconstruction of an *in vivo* juxtacellularly labeled AVP+ magnocellular neuron. The soma and dendrites were represented in black and axonal segments were represented in red. AVP-containing nature was ascertained by AVP immunoreaction **(E1)**. The soma gave rise initially to two short thick primary dendrites, which branched proximally. The main axon coursed laterally passing the fornix (fx), turned ventro-caudally toward the posterior pituitary gland. Two main collaterals emanated from this axon **(E2,E3)**. The first collateral **(E2)** coursed dorsomedially joining the *stria medularis* (sm). Neurobiotin labeled processes were found inside the lateral habenula **(E4)**. [The panel **(E)** was modified from (Hernández et al., 2015)]. 3V: third ventricle; Opt: optic tract; SCN: suprachiasmatic nucleus; PaLM: paraventricular lateral magnocellular. Scale bars: **A, C′, C′ a–b**, and **D′, D′ a–b**: 100 μm; **B′ a–b**: 20 μm; **E**: 250 μm; **E1**: 20 μm, and **E4**: 50 μm.

VP containing cells located in SON and PVN have large somata and are commonly known to project to the posterior pituitary gland where they release the nonapeptide vasopressin, which is critical for cardiovascular functions and hydro-electrolytic homeostasis (Bargmann and Scharrer, 1951). Using Fluoro-Gold (FG) iontophoretical injection into the medial subdivision of LHb (Figure 3A), we found some VP+ cells in the suprachiasmatic nuclei (SCN), which were retrogradely labeled (Figure 3Bs). In contrast, abundant VP+ magnocellular neurons in both PVN and SON were retrogradely labeled (Figures 3Cs,Ds). In a recently published paper (Hernández et al., 2015), we reported *in vivo* juxtacellularly labeled individual VP+ magnocellular morphology: all the reported neurons possessed multiaxons and axon collaterals projecting to intracerebral structures other that neurohypophysis. Figure 3E is one example: an *in vivo* juxtacellularly labeled magnocellular VP+ neuron (Figures 3E,E1) emitted an axonal collateral (Figure 3, point E2) which coursed dorso-rostro-medially to join the stria medullaris (sm). A neurobiotin-labeled axon segments were found in the medial division of LHb.

### WD24 led to reduced freezing and immobility during innate fear processing and behavioral despair respectively

From the observation of VP region-specific innervation and the hypothalamic magnocellular origin of this innervation, came the question about the psychomotor consequences of the integrative role of this pathway while coping with internal (homeostatic) and external adversities. LHb activation has been shown to induce aversive learning and promote passive stress copping strategies, seen experimentally as an increased freezing behavior during innate fear processing and immobility accounts/time during bahavioral despair(Pobbe and Zangrossi, 2008; Bowen et al., 2013; Gill et al., 2013). For these reasons, we devised two simple physiological tests placing the rats in psychological and physical live-threatening conditions: (1) assessing the innate fear processing using the exposure to a live cat (Blanchard et al., 1975), and (2) assessing behavioral despair using a modified version of the FST (Porsolt et al., 1977). Positive and negative motivational valence representations were correlated with freezing vs. rearing/climbing/displacement and immobility vs. climbing during cat exposure and FST, respectively. In both cases one group of rats underwent water deprivation for 24 h (WD24). It is worth mentioning that WD24 produces a less than 3% of increase in rat plasma osmolarity but till to seven-fold increase of VP plasma concentration (Dunn et al., 1973).

**Figure 4:**
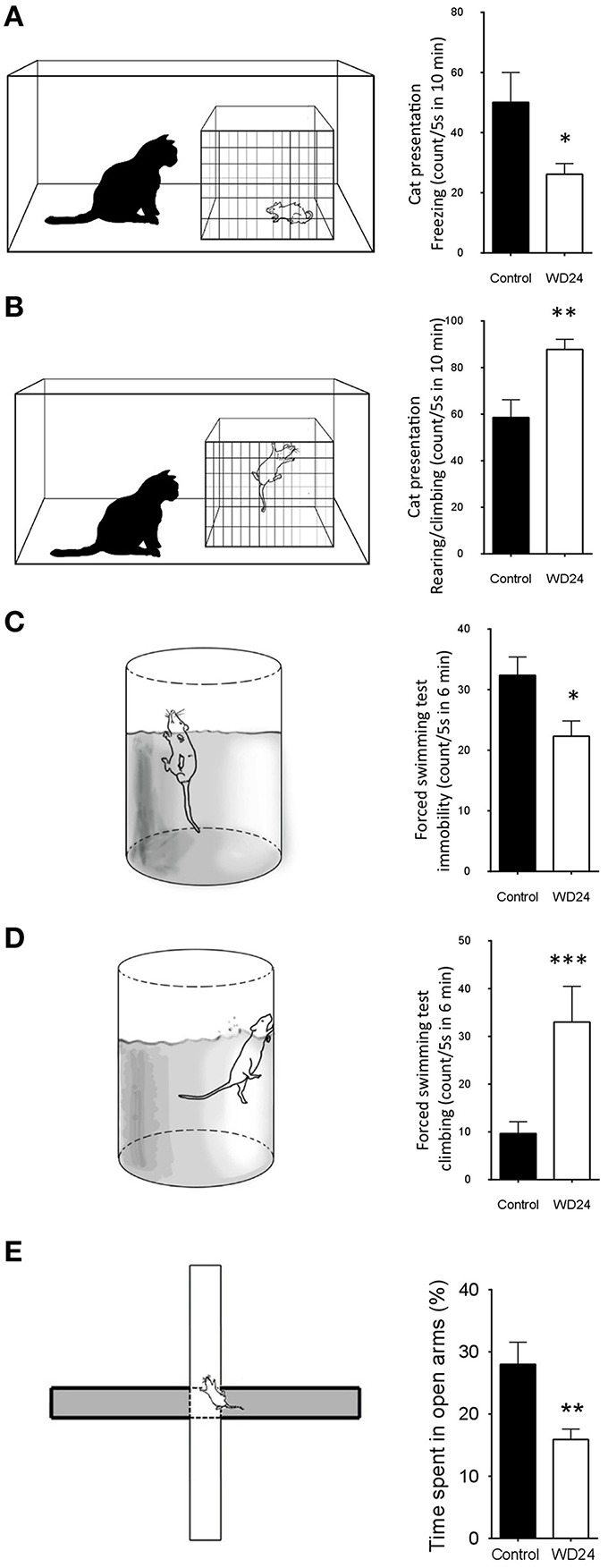
**Twenty-four hours of water deprivation (WD24) promoted active stress coping during innate fear processing (cat exposure) and behavioral despair (forced swimming test, FST)**. WD24 is a potent physiological stimulus to increase metabolic activities of AVP containing magnocellular neurosecretory neurons in SON and PVN. Upon cat exposure, rats expressed innate fear-related passive (freezing), and active (rearing, climbing, and displacement) behaviors **(A,B)**. Rats from WD24 group showed significant reduction of freezing counts **(A)** and increase of climbing and rearing behaviors **(B)**. Similar observations were obtained during FST for behavioral despair **(C,D)**. For locomotor control, we performed the elevated plus maze (EPM) test to both groups **(E)**. The WD24 rats showed normal locomotion patterns but reduced percentage of time spent in open-arms. (Mean ± SEM, ^*^p < 0.05, ^**^p < 0.01, ^***^p < 0.001).

The effects of WD24 on the passive stress coping strategies (freezing) or active stress coping strategies (rearing/climbing/displacement), displayed when exposed to a live predator, are shown in (Figures 4A,B). The WD24 group showed a significant reduction in freezing counts (Ctrl: 50.1 ± 9.99 vs. WD24: 26.2 ± 3.5, *n* = 12, *p* < 0.05) and a significant increase in the number of active escaping (rearing/climbing/displacement) counts (Ctrl: 58.6 ± 6.3 vs. WD24: 87.8 ± 4.3, *n* = 12, *p* < 0.001). The water deprivation effects on swimming behavior during the FST are illustrated in Figures 4C,D. In the WD24 group there was a significant decrease in the immobility episodes (Ctrl: 32.3 ± 3.0 vs. WD24: 22.3 ± 2.5, Ctrl *n* = 11, WD24: *n* = 12, *p* < 0.05) and a significant increase in the climbing episodes (Ctrl: 9.7 ± 2.4 vs. WD24: 33.1 ± 2.5, Ctrl *n* = 11, WD24: *n* = 12, *p* < 0.001).

For locomotor control, we performed the elevated plus maze (EPM) test to both groups. The WD24 rats showed normal locomotion patterns but reduced percentage of time spent in open-arms, from 28 ± 3.56% to 15.91 ± 1.67% (*n* = 5, *p* < 0.01) (Figure 4E). It is interesting to note that the WD24 group showed more cautious movements in the open-arms than the control group, i.e., sticking only their head out of the central square on more occasions, rather than walking on the open-arms.

### WD24 induced spatial neuronal activation pattern coincided with VP+ fiber distribution; most fos+ cells were GABA+ neurons

**Figure 5:**
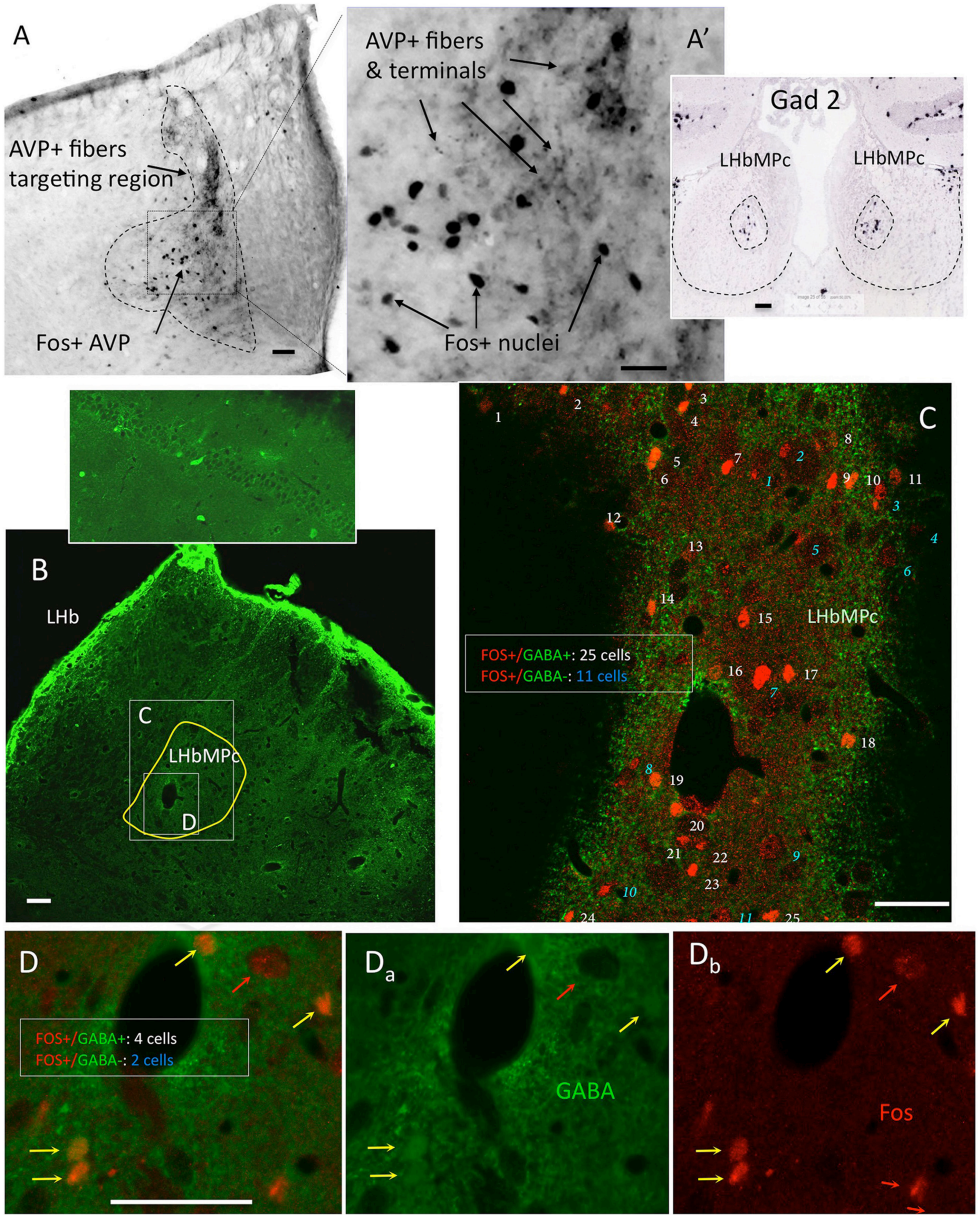
**Twenty-four hours of water deprivation induced strong Fos expression in LHbMC; most of the activated neurons were GABA immunopositive. (A)**. WD24 produced a spatial pattern of neuronal activation which coincided with AVP+ fiber distribution, i. e. the medial central nucleus of the lateral habenula LHbMC. Inset shows Gad2 *in situ* hybridization pattern in LHbMC (obtained from the Allen Institute for Brain Science (http://mouse.brain-map.org/experiment/show/79591669). Panels **(B–D)** confocal images for coronal sections of habenula with double immunofluorescence showing that most of the Fos+ cells were GABA+ neurons. White consecutive numbers and yellow arrows indicate Fos+/GABA+ neurons, whereas blue consecutive numbers indicate Fos+/GABA- neurons. Panels **(D_a-b_)** show the magnified squared region of **(B)**, where 6 Fos+ nuclei are found, 4 of them had GABA+ cytoplasms (yellow arrows) and 2 had GABA negative cytoplasms (red arrows). Scale bar: 50 μm.

After observing that WD24 decreased the freezing behavior, the immediate early gene fos protein product Fos was studied using immunohistochemistry (IHC), together with VP and GABA immunoreactions. The expression of Fos was greatly increased in LHbMC subnucleus, where VP+ fibers were densely distributed (see Figures 5A,A′). Double Fos/GABA immunofluorescence revealed that most of the neurons activated by WD24 were GABAergic (Figures 5B–D).

### LHbMC host a distinct population of GABAergic interneurons; the presence of vasopressin increase their excitability

**Figure 6:**
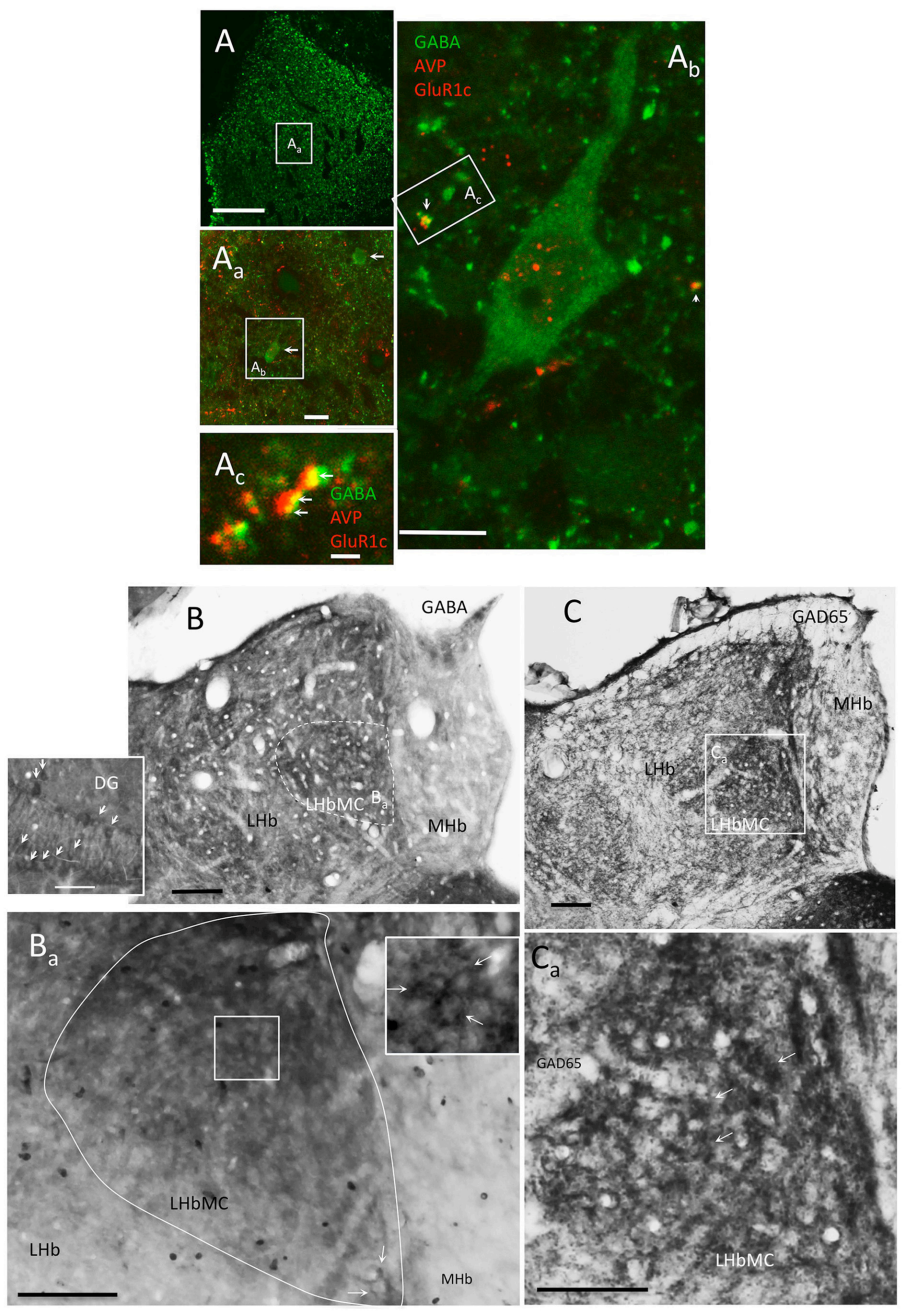
**LHbMC host a distinct population of GABAergic neurons. (A, A_a_)** Confocal images showing the location of some GABA+ neurons (arrows) in combination with AVP and GluR1c immunofluorescence reaction, using high glutaraldehyde fixation. **(A_b_)** An enlargement of one GABA positive neuron and its relation with the other two antigens. Notice that the antibody for GluR1c produced some nuclear background labeling. **(A_c_)** Confocal image of 700 nm thickness of a dendritic segment of the cell **(A_b_)**_._ Notice that some labeled dendrites (green process) contained postsynaptic glutamate receptor type 1 (GluR1c, yellow points indicated by arrows) in juxtaposition to the AVP axon terminals (red process). **(B,C)** GABAergic neurons were mainly observed in lateral habenula medial central subnucleus (LHbMC) through immunoreaction against GABA and GAD65, respectively, in brain tissues posterior to 6 h of intra-habenular colchicine injection which enhanced GABA and GAD65 labeling (Ribak et al., 1978). The inset is for reference immunohistochemistry reaction of dentate gyrus (DG) of the same brain section. Notice the similarities of labeling patterns between DG GABAergic neurons (arrows in inset of **B**) and the habenular-labeling pattern (indicated by arrows in the inset of **B**_a_). **(C)** Immunoreaction on the same brain tissue, using antibody against GAD65. **(C**_a_) Magnification centered at the medio-central subnucleus of the lateral habenular complex, some labeled cells are indicated with white arrows. Scale bars: **A**: 100 μm; **A**_a_: 20 μm; **A**_b_: 10 μm; **A**_c_: 1 μm; rest: 50 μm.

Although GABAergic interneurons are the main source of synaptic inhibition in the cortex, the existence of this kind of neurons in the epithalamic complex has been a matter of debate. Several reports have argued for the absence of GABAergic neurons inside LHb, although transcripts of gad2 (Wagner et al., 2016) and gad1 (supplementary information, Figure S4) were found in the LHbMC by using conventional *in situ* hybridization and RNAscope, respectively. GABA or GAD65 or GAD67 somatic labeling by conventional IHC has been a troublesome issue for a long time. We took the challenge with a wide range of antibodies, and a modified IHC protocol. By using two highly purified antisera against GABA-glutaraldehyde-BSA (Millipore MAB319, anti-GABA, clone 5A9, and Sigma A0310, clone GB-69) and GAD65/67 and intra-habenular injection of colchicine, we observed that there is a unique population of GABA/GAD containing neurons inside the rat LHbMC (Figure 6). Using high glutaraldehyde fixation, some GABA containing neuron's somata and proximal dendrites could be clearly labeled (Figures 6A_a_, A_b_). Some labeled dendrites making close contacts with VP+ fibers contained postsynaptic glutamate receptor type 1 (GluR1) in juxtaposition to the VP axon terminals (Figure 6A_c_, arrows) suggesting the existence of glutamatergic synapses. To test whether there are GABAergic neurons in the LHbMC we modified the conventional immunohistochemistry procedures: (1) by microinjection of colchicine into LHbMC 6 h previous to the perfusion/fixation which has proved to increase the GABA/GAD somatic labeling (Ribak et al., 1978); (2) by using high glutaraldehyde (0.1%) fixative; (3) by reducing the triton-100x usage (0.03%) for GABA and GAD IHC. Figures 6B,C show that there were abundant GABA and GAD somatic labelings, similar to the GABAergic neurons in the hippocampal dentate gyrus, in the LHbMC region.

Together with the EM evidence that VP+ axon terminals synapse (Gray type I) onto habenular neuron's dendrite (Figures 2C,D), it was clear that the GABAergic neurons are one of the postsynaptic targets of VP+ axons. When looking for identities of GABAergic neurons, we assessed the GABA expressing neurons immunoreactive for metabotropic glutamate receptor 1α (mGluR1 α). We found that most of the somatic membranes labeled by mGluR1α co-expressed GABA, in the medial division of the LHb (supplementary information Figure S2A). Moreover, we found a strong contact relationship between mGluR1α expressing dendrites and VP+ axons (Figure S2B).

We sought to determine whether VP influences the electrical activity of LHbMPc neurons by performing whole-cell patch-clamp recording in acute coronal brain slices and applying VP (10 nM) to the recording chamber. VP induced a sustained increase of firing rate in 50% of the recorded neurons (Type I, Figure S3A). *Post-hoc* anatomical analysis of 3 cells with axons clearly conserved and labeled with biotin showed proximal branching in the medial subregion and a long axon extending to the lateral subregion of LHb. We identified the chemical markers of these cells as follows: GAD65+ (soma and axon), vGAT+ (axon) and mGluR1a+ (soma and dendrite). Another 30% of recorded cells decreased their firing rate upon VP application (named type II) (Figure S3B). The type II somata were all bipolar shaped with a long single axon projecting to fasciculus retroflexus (fr). The soma and dendrites of these neurons were positive to GIRK1, a G protein coupled inward rectified potassium channel and their axons expressed vGluT2. VP bath application reduced significantly the input resistance of this group of neurons. The remaining 20% of the recorded cells in the LHbMC exhibited no evident response to VP application (see supplementary information Figure S3).

### *In vivo* juxtacellular labeled individual LHbMC cells confirmed the interneuron identity of the GABA cells

**Figure 7:**
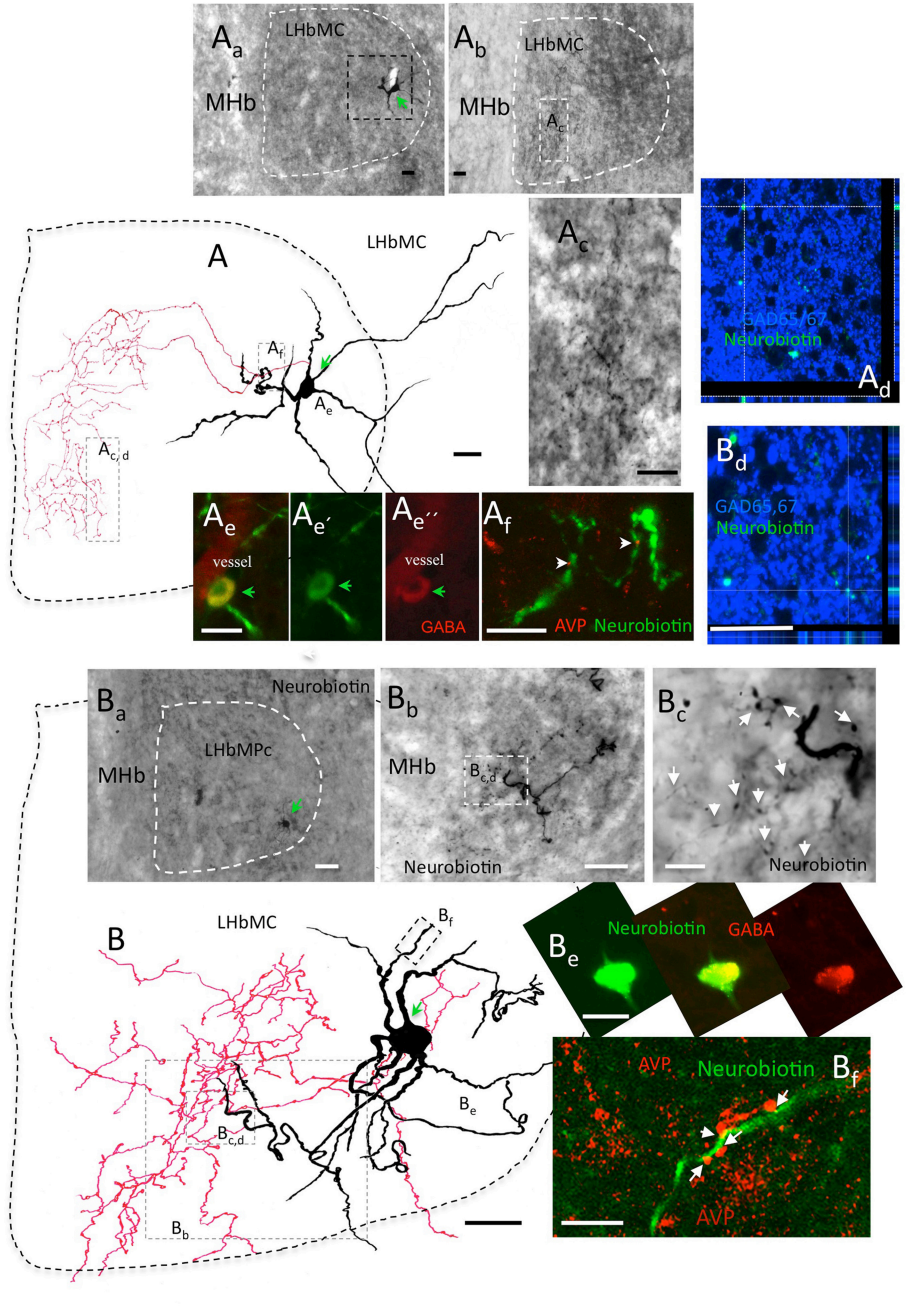
***In vivo* juxtacellular labeled individual LHbMC cells confirmed the interneuron identity of GABA cells. (A)** : a camera lucida reconstruction of a juxtacellularly labeled cells superimposing 8 coronal sections of 70 μm, respectively. **(A_a_)** The soma was located in the lateral part of the LHbMC, around Bregma (Br) −3.84 mm, according to Rat Brain Atlas (Paxinos and Watson, 2006). **(A_b_)** Labeled axons were concentrated around the rostro-caudal coordinate Br –3.48 mm. **(A_c_)** Is amplification of **(A_b_)** squared region. **(A_e_)** and **(A_d_)**: The soma and axon terminals were tested immunopositive to GABA and Gad 65/67, respectively. **(Af)**: A close contacting relationship was observed between the neurobiotin labeled dendrite and VP+ axons. **(B)**: Another example of *in vivo* juxtacellularly labeled GABAergic interneuron. Camera lucida reconstruction was obtained from 10 coronal sections of 70 μm, respectively. **(B_a_)** Soma **(B)** was located in the lateral part of the LHbMC, around Br −3.72 mm. **(B_b_)** The main axonal ramification was found in sections caudal to the soma. **(B_c_)** is an amplification of the squared region of **(B_b_)**. The axon was tested to be GAD65/67+ with confocal microscopy **(B_d_)** while the soma was GABA+ **(B_e_)**. This cell's dendrites were also contacting AVP+ axons **(B_f_)**. Scale bars: 20 μm.

After finding this GABAergic neuronal population, we further hypothesized that there is a GABAergic interneuron microcircuit within the habenular complex modulating the functional output of the LHb. We first examined this hypothesis by performing *in vivo* juxtacellular labeling experiment targeting the cells in the LHbMC. Here, we present two *in vivo* labeled GABAergic neurons that possessed short and extensively branched axons inside the LHbMC. Figure 7 shows camera lucida reconstructions of the two labeled cells superimposing 8 and 10 serial coronal sections of 70 μm, respectively. The soma A was located in the lateral part of the LHbMC, around Bregma (Br) −3.84 mm, according to Rat Brain Atlas (Paxinos and Watson, 2006) (Figure 7A_a_), whereas the labeled axons were concentrated in the rostro-caudal coordinate Br −3.48 mm (Figures 7A_b_–A_d_). The soma and axon terminals tested immunopositive to GABA and Gad 65/67, respectively (Figures 7A_e_,A_d_). A close contacting relationship was observed between the neurobiotin labeled dendrite and VP+ axons (Figure 7A_f_). The soma B was located also in the lateral part of LHbMC but rostral to the soma A (Br −3.36 mm, Figures 7B, B_a_). This cell had an unusual extensive and winding dendritic arborization. The soma was tested to be GABA+ and axons GAD65/67+. The main axonal ramification was found in the section around Br −3.72 mm. This cell's dendrite was also found contacting VP+ axons (Figure 7B_f_). It is notable that the soma locations of these two cells were quite far from the main branching sites (approximately 400 μm in rostro-caudal axis).

### Differential neuronal activation of LHb by cat exposure, FST and WD24+Cat exposure and WD24+FST suggested that the VP/glutamate pathway played a role in enhancing the LHbMC neuronal population's excitability

**Figure 8:**
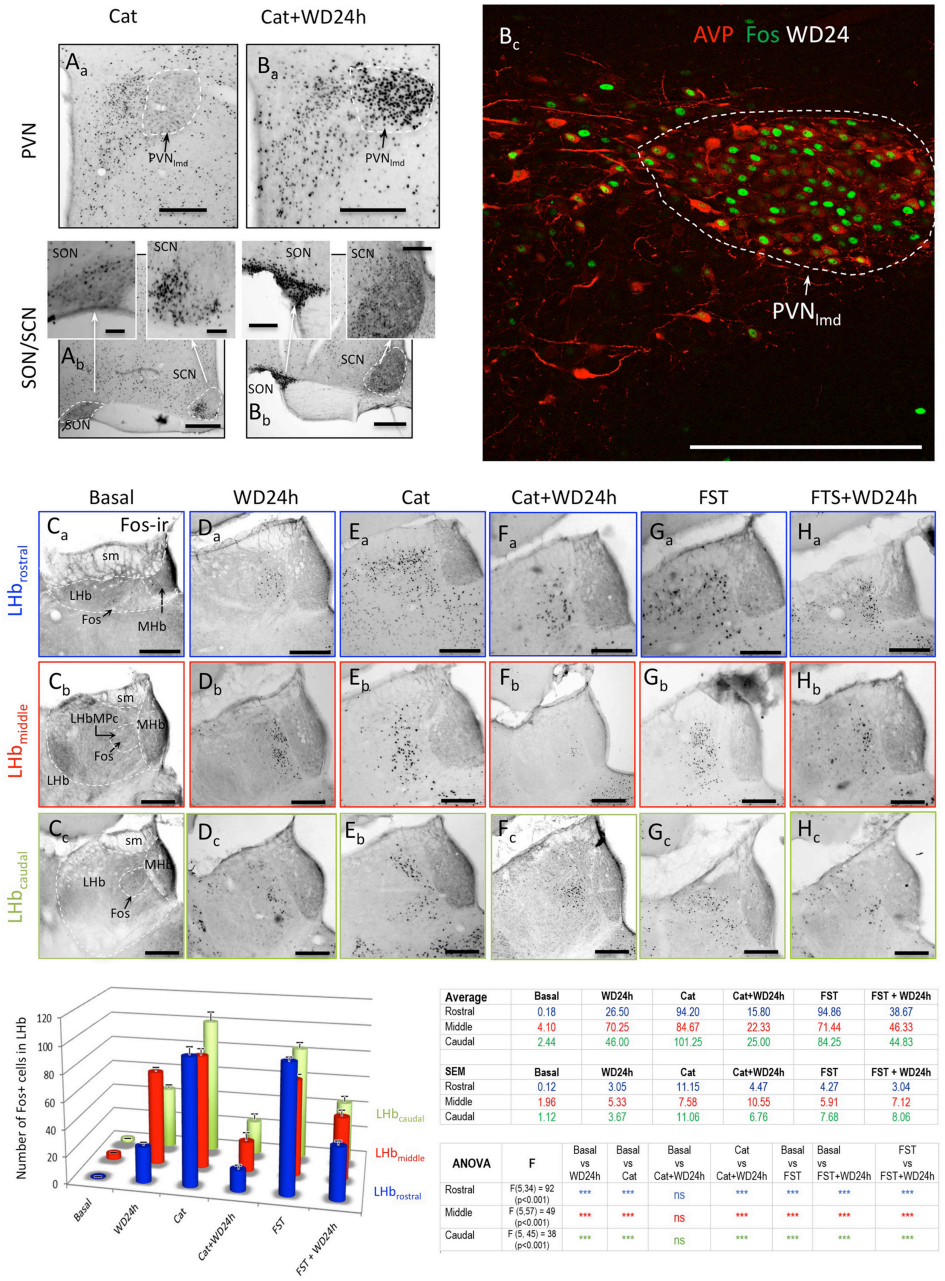
**Differential neuronal activation of lateral habenula by cat exposure or forced swimming test (FST) in rats with or without 24 h of water deprivation (WD24) suggested AVP-glutamate pathway activates habenular internal inhibitory mechanisms**. Representative photomicrographs of Fos expression patterns: panels **(A)** and **(B)** show the Fos expression patterns in the hypothalamic AVP containing paraventricular (PVN), supraoptic (SON) and suprachiasmatic (SCN) nuclei, as a consequence of either cat exposure alone **(A_a_,A_b_)** or cat exposure+WD24 **(B_a_,B_b_)**. Panel **B_c_** shows the Fos expression in PVN after WD24. PVNlmd: lateral magnocellular division of PVN; PVNmpd: medial parvocellular division of PVN; **(C_a-c_–H_a-c_)**: representative photomicrographs of Fos expression corresponding to each group at each habenular rostro-caudal levels (lettered and framed with the same color code of the histogram). Color-coded histograms and statistical table show Fos+ nuclear counting in whole habenular sections, sampled at three rostro-caudal levels, namely, the rostral (blue), the middle (mid, red) and the caudal (green) of 6 experimental groups: basal (*n* = 4), WD24 (*n* = 4), cat exposure (*n* = 8), cat exposure+WD24 (*n* = 8), FST (*n* = 5), FST + WD24 (*n* = 7). Quantitative results and significance levels are provided in the adjacent table. Scale bars: 100 μm.

The observation of GABAergic interneurons in the LHbMC and their branching patterns prompted us to study the immediate early gene fos protein product Fos expression at three rostro-caudal levels in 6 different groups: basal, WD24, cat exposure, cat exposure+WD24, FST, FST+WD24 (*n* ≥ 4). Figure 8A showed the Fos activation patterns, due to cat exposure, in the three main vasopressin containing hypothalamic nuclei: PVN, A_a_, with low expression in the PVN lateral magnocellular division (PVN_lmd_) and high expression in the medial parvocellular division (PVN_mpd_); there was some Fos expression in the SON and in the ventrolateral part of the SCN there was strong activation (this expression pattern was probably due to the circadian time of the rat); the dorsomedial part was mainly inactive (Figure 8A_b_ and insets). Cat exposure +WD24 activated both the PVN_lmd_ and the PVN_mpd_ (Figure 8B_a_)_._ The SON was highly activated so was the SCN (Figure 8B_b_ and insets). It is worth mentioning that the WD24 *per se* strongly upregulates the magnocellular vasopressin containing neuron's metabolic activity with an increase of VP plasma concentration from 3 to 7 fold (Dunn et al., 1973; Zhang et al., 2010). Figure 8B_c_ showed that WD24 alone induced Fos expression in almost all the magnocellular VP neurons. Habenular patterns of neuronal activation measured by Fos+ nuclear accounts were strikingly different between rats with and without WD24, after exposing to cat or to the FST. Under basal conditions, the habenular complex showed low Fos expression in the three rostro-caudal levels, (Figures 8C_a-c_ and histogram); WD24 activated mainly a selective neuronal population in the medio-central and medio-ventral parts of the lateral habenula (Figures 8D_a-c_ and histogram); cat exposure (Figures 8E_a-c_ and histogram) or FST (Figures 8G_a-c_ and histogram) produced vast and diffuse neuronal activation shown as an increase of global Fos expression, whereas WD24 before the behavioral tests reduced the neuronal activation levels significantly (Figures 8F_a-c_, Ha–c and histograms “Cat+WD24” and “FST+WD24”). The quantitative data for each group (media ± SEM) and ANOVA results are presented in the table next to the histograms in Figure 8. These significant reductions of global Fos+ nucleus counts in three rostro-caudal LHb levels (rostral, middle, and caudal) indicated that WD24 exerts potent suppression of the functional output of the lateral habenular complex.

## Discussion

In this study, we investigated vasopressinergic innervations in the rat habenular complex and its functional implications. We show that a direct VP-containing glutamatergic pathway, from VP-MNNs' axon collaterals, synaptically targeted the sub-nuclei of the medial division of LHb (LHbM), region identified as containing GABAergic interneurons, demonstrated through *in vivo* individual cell labeling and immunohistochemistry. Physiological up-regulation of the VP-MNNs system by WD24 was associated with suppression of LHb functional output, which correlated with active stress coping showed as decreased freezing and immobility during innate fear and behavioral despair assessments. These experiments suggest that this VP-glutamate pathway from the hypothalamic thirst-hydro-electrolytic homeostatic circuit participates in the motivation encoding under multifaceted stressful situations, probably through the activation of a GABAergic microcircuit within the habenular complex.

Stress coping is essential for survival. Strategies to manage stress range from active to passive—the former prepares the animals for a successful control of threats by performing goal-directed movements (e.g., *fight* or *flight*), while the latter, expressed as psychomotor deficit (e.g., *freezing or immobility*), is used when aversively perceived encounters are judged not to be controlled by fight or flight (Engelmann et al., 2004). It seems straightforward that the decision-making is intimately linked to the neuroendocrine states. However, beyond the hypothalamus-pituitary-adrenal (HPA) axis, little is known about involvement of other functional circuits. Recent advances in this topic include the effect of hunger on fear extinction (Verma et al., 2016), hypernatremia on HPA inhibition and post-stress recovery (Krause et al., 2011), and the discovery of VP containing MNNs that project to the hippocampus (Cui et al., 2013; Zhang and Hernández, 2013), which are likely to influence spatial learning (Hernández et al., 2012), anxiety (Zhang et al., 2012) and aggressive behavior (Pagani et al., 2015).

Our first novel finding is the discovery of the origin of VP innervations in the lateral habenula by both Fluoro-Gold retrograde labeling and *in vivo* juxtacellular anterograde labeling. In a recently published paper (Hernández et al., 2015), we characterized extra-neurohypophyseal axonal projections from individual vasopressin-containing magnocellular neurons in the rat hypothalamus. We showed that the VP-MNNs possessed multiple axon-processes (Hernández et al., 2015) and the long-range non-neurohypophysial projections is a more common feature of this type of neuron, than an “occasional” phenomenon as previously thought to be, suggesting that the magnocellular VP-glutamatergic non-canonical pathways found here may constitute the central motivational circuits activated under multifaceted stress coping.

Our second key finding is that at the electron microscopy level, VP+ dense-core vesicles (dcv) co-localized with small-clear vesicles at the active synaptic zone, with some of them docked at the presynaptic membrane, suggesting that VP could act as a direct synaptic transmission modulator via a yet-unknown mechanism. It has been identified so far that VP binds mainly to three distinct G-protein coupled receptors: (i) vasopressin V1A receptors, that trigger phospholipase-C C (PLC) activation and calcium mobilization; (ii) vasopressin V1B receptors, that are also coupled to PLC and are found mainly in the anterior pituitary and hippocampus CA2 region; (iii) vasopressin V2 receptors, that are coupled to adenylyl cyclase, and are present in the kidney (Barberis et al., 1998; Schoneberg et al., 1998; Thibonnier et al., 1998; Birnbaumer, 2000). Although low expression of both V1a and V1b receptor transcripts have been observed in the lateral habenular region by *in situ* hybridization (Figure S3D and Allen Brain Atlas, Allen Institute, http://mouse.brain-map.org/experiment/show?id=78153154, http://mouse.brain-map.org/experiment/show?id=79488955), the presence of receptors and their subregional and synaptic/extrasynaptic distribution in lateral habenular complex have remain unclear. One of the reasons for which this information is still obscure is due to lack of trustworthy antibodies and suitable techniques to identify the presence of VP receptors in the postsynaptic membrane. Nonetheless, the synergistic effects of vasopressin on fast and long-lasting glutamate-mediated excitatory synaptic events have been extensively reported (Muhlethaler et al., 1982; Chen et al., 1993; Kombian et al., 2000; Chepkova et al., 2001; Dubrovsky et al., 2003). In particular, several VP metabolites have been identified to greatly contribute to the observed VP effects in the central nervous system. In contrast to VP, very low doses of those VP metabolites (e.g., 1/1000 of pharmacological dose of VP), which have no peripheral effects, have been found to induce changes in the CNS (Burbach et al., 1983; Dietrich and Allen, 1997; Fujiwara et al., 1997; Reijmers et al., 1998). These data favor the hypothetical existence of a separate “receptor” for the VP metabolites distinct of the classical G-protein coupled VP and oxytocin receptors, although its identity has remained obscure. Another hypothetical mechanism to explain the experimental evidence mentioned above, including the presence of VP+ dcvs in the presynaptic membrane observed under electron microscopy we are reporting with this work, is that the vasopressin metabolites interact directly with postsynaptic proteins facilitating the glutamatergic transmission. Peptide-protein interactions are prevalent in the living cell and form a key component of the overall protein-protein interaction network (London et al., 2012). Hence we would speculate that the interaction between VP-metabolites and the postsynaptic glutamate receptors underlies this glutamate transmission-synergic effect of brain VP. The study of these possible mechanisms is a major challenge to understand how brain VP system generates and regulates diverse central functions.

The presence of intrinsic GABAergic neurons in the rat LHb had been suggested more than 30 years ago (Gottesfeld et al., 1980, 1981; Belin et al., 1982). However, it has been generally accepted that the LHb lacks inhibitory interneurons and that the strong GABAergic innervation observed under IHC is presumably coming from long-range projections (Meye et al., 2013). As a consequence, less attention has been paid to the contribution of intrinsic inhibitory interneurons on habenular physiology. However, this provisional conclusion could be due to the technical difficulties to label the soma and dendrites of GABA neurons by conventional IHC methods, although both GAD65 and GAD67 mRNA have been seen using either conventional *in situ* hybridization methods (Brinschwitz et al., 2010; Wagner et al., 2016) or the newly available RNAscope method (Advanced Cell Diagnostic, CA; Figure S4). By modification of the fixation and IHC procedures, and by intrahabenular injection of colchicine, which enhanced the GABA somatic labeling (Ribak et al., 1978), we have seen clearly the cytoplasmatic presence of either GABA or GAD65/67 in the LHbMC. This observation is further unequivocally corroborated by *in vivo* and *in vitro* labeled GABAergic neurons whose axonal branches were largely seen inside the LHbMC, particularly in the parvocellular subnucleus of the central part of the LHb (LHbMPc). Long term metabolic activation of hypothalamic VP-glutamate pathway due to WD24 24 h (see Figure 8B_c_, striking Fos expression in almost all VP+ neurons inside the PVN_lmd_ and Figures 5C,D, that most Fos+ nuclei co-express GABA in the LHbMC) reduced significantly the Fos expression in whole habenular complex when rats were subjected to life-threatening situations and correlated with the reduced freezing and immobility behaviors. These results suggest that the pathway involving VP-MNNs projections to the LHb could be a feed-forward inhibition arrangement, i.e., numerically not-so-abundant VP containing excitatory inputs selectively activate local inhibitory interneurons, which, in turn, suppress the functional output of the LHb.

We aimed to investigate the influences of an internal stressful condition (i.e., thirst caused by 24 h water deprivation, in which the hypothalamic VP magnocellular system is greatly up-regulated) on the life-threatening stress response (live-predator exposure and drowning). We would like to call this as a “meta-stress” coping. Our results suggest that WD24 enhance the motivational behavior (escape) and down-regulate the passive stress coping (freezing). This observation was correlated with differential Fos activation patterns in the LHb. Concerning the forced swim test (FST), we modified the classical setting to a simple one-time exposure, aiming to answer our research question—can thirst (an internal stressful condition) increase the motivational behavior toward survival when the animal is facing a life-threatening condition (a multifaceted adversity). An acute episode, when the animal faces the threat of drowning for the first time in this setting (no adaptation of the inescapability chance has been given), the emotional component of the classical setting is minimized. Hence, we consider that this modified FST infers the animal internal motivational state—“escape or wait to die” and it is valuable to assess an acute active vs. passive stress response. LHb activation has been shown to induce aversive learning and promote passive stress copping strategies, seen experimentally as an increased freezing behavior during innate fear processing and immobility accounts/time during behavioral despair (Pobbe and Zangrossi, 2008; Bowen et al., 2013; Gill et al., 2013). The results suggest that AVP-MNNs to LHb pathway could contribute in promoting motivational behavior during a multifaceted stress coping.

We want to emphasize that an important strength of this study is that the behavioral tests were performed under physiological conditions. The hypothalamic magnocellular VP system possesses some unique advantages to study the behavioral consequences of the activation of a given neuronal set. This system can be potently up-regulated under thirst, in a relatively selective way (Figure 8B_c_), even before a significant disruption of the body's water homeostasis occurs [for instance, WD24, in which the plasma osmolarity increases around 3% while the plasma VP concentration increases 7 fold (Dunn et al., 1973)]. This feature offers a valuable opportunity to study the behavioral consequence of the activation of a selective neuronal population strictly within the physiological circuitry controls—no genetic modification, viral infection, artificial over-stimulation or pain caused by invasive procedures are involved.

From these data it could be further hypothesized that an intrinsic GABAergic microcircuit exists for the fine control of habenular functional output, a region considered to be part of the limbic system. During the preparation and reviewing of this manuscript a publication appeared (Myers et al., 2015) reporting evidence of limbic regions controlling posterior hypothalamic GABAergic microcircuits, which in turn suppress the excitatory output to the HPA axis, reducing anxious behavior and social withdrawal. The data from our study suggest that hypothalamic homeostatic circuits could modulate the GABAergic microcircuit within the LHb, a limbic region, to promote motivational behaviors, echoing with the previous work. Together, these novel results provide a beautiful example of how distinct brain regions work in concert to produce mental and behavioral actions to promote survival.

Further studies on the synaptic organization and the interactions with other transmitter systems, in particular, the TH+ and SerT+ fiber systems in the habenula, will provide a more complete view of brain's physiological circuits for motivational behaviors.

## Author contributions

Conceived and designed the experiments: LZ, VSH. Performed the experiments: LZ, VH, EV, and FC. Analyzed the data: LZ, VSH, EVJ, FKC, and RAB. Contributed equipment/reagents/materials/analysis tools: LZ and RAB. Wrote the paper: LZ and VH. Revised the manuscript critically for important intellectual content: all the authors.

### Conflict of interest statement

The authors declare that the research was conducted in the absence of any commercial or financial relationships that could be construed as a potential conflict of interest. The reviewer AD and handling Editor declared their shared affiliation, and the handling Editor states that the process nevertheless met the standards of a fair and objective review.

## Supplementary material

Figure S1 **Representative photomicrographs of relevant neurochemical marker expression in habenula in relation to vasopressin innervation**. Boxed regions were subsequently magnified. AVP: arginine vasopressin **(A)**; TH: tyrosine hydroxylase **(B)**; SerT: serotonin transporter **(C)**; SOM: somatostatin **(D)**; ENK: encephalin **(E)**; SP: substance P **(F)**; CR: calretinin **(G)**; CB: calbindin **(H)**; G-protein-activated inward rectifying potassium (GIRK 2) **(I,I′)** and G-protein-activated inward rectifying potassium (GIRK 1) **(J)**. LHbMC: medio- central subnucleus of lateral habenula, circumscribed region. Scale bar: 100 μm.

Figure S2 **Metabotropic glutamate receptor 1**α** (mGluR1**α**) expressing GABAergic neurons were among the vasopressinergic innervation's targets. (A)** Confocal images of immunohistochemical labeling for mGluR1α (red) and GABA (green), showed that numerous GABAerigic neurons, in the medial part of the lateral habenula (LHbM), had their somata and dendrites decorated by mGluR1a (arrows). **(B)** Confocal images of immunohistochemical labeling of AVP (green), mGluR1α (red) in LHbM. Notice that there was a strong-contacting relationship between AVP axons and mGlu1Rα expressing dendritic segments. Scale bar: 20 μm.

Figure S3 ***In vitro* electrophysiological responses of neurons from the medio-central subnucleus of the lateral habenula (LHbMC) to 10nM AVP bath application: morphology and chemical identities**. Whole-cell patch-clamp recording on the medial part of the lateral habenula (LHbM) neurons in acute coronal brain slices applying AVP (10 nM) to the recording chamber induced differential electrophysiological response: about 50% of recorded neurons increased their firing rate. Chart in panel **(A)** depicts the location of 11 cells, which were successfully labeled and immunoreacted as GABAergic neurons in the medio-central part of the lateral habenula (LHbMC). **(A_a_)**: a sample of reconstructed type I neuron with axons branching extensively near the soma (red lines in **A_a_** and green biocytin labelling in **B_a_**, indicated by double blue arrowheads), expressing GAD65 in soma and axons, and terminals [purple double arrowheads **(B_b_)**]. The axon was detected to be VGaT+ (Cs). **(D)**: by *in situ* hybridization using the RNA scope multiplex method, we found that numerous cells in this region co-express mRNAs for Gad1 (green) and vasopressin receptor type 1a (V1a, blue). Another 30% of recorded cells decreased their firing rate upon AVP application. **(E)**: camera lucida reconstruction of a sample neuron. Its input resistance was reduced significantly upon VP bath application (histogram). Some of the cells were well-labeled with somata showing bipolar shape, with extensive dendritic arborization from both poles and a single long axon projecting to the fasciculus retroflexus. The dendrites had sparse spines **(E_b_)**. The soma and dendrite of these neurons were positive to GIRK1 (**E_a_** and **E_c_**). A G protein coupled inward rectified potassium channel and their axons expressed vGlut2 **(E_d_)**. Scale bars: 10 μm.

Figure S4 **RNAscope ISH Assays confirmed the presence of Gad67 RNA in rat LHbMC**.

Table S1 **Antibody information, related to Material and Method**.
