# Supplementary figures and images for: Thirst Is Associated with Suppression of Habenula Output and Active Stress Coping: Is there a Role for a Non-canonical Vasopressin-Glutamate Pathway?
Source: Front Neural Circuits. 2016 Mar 31;10:13. doi: 10.3389/fncir.2016.00013 (PMC4814529; doi:10.3389/fncir.2016.00013)

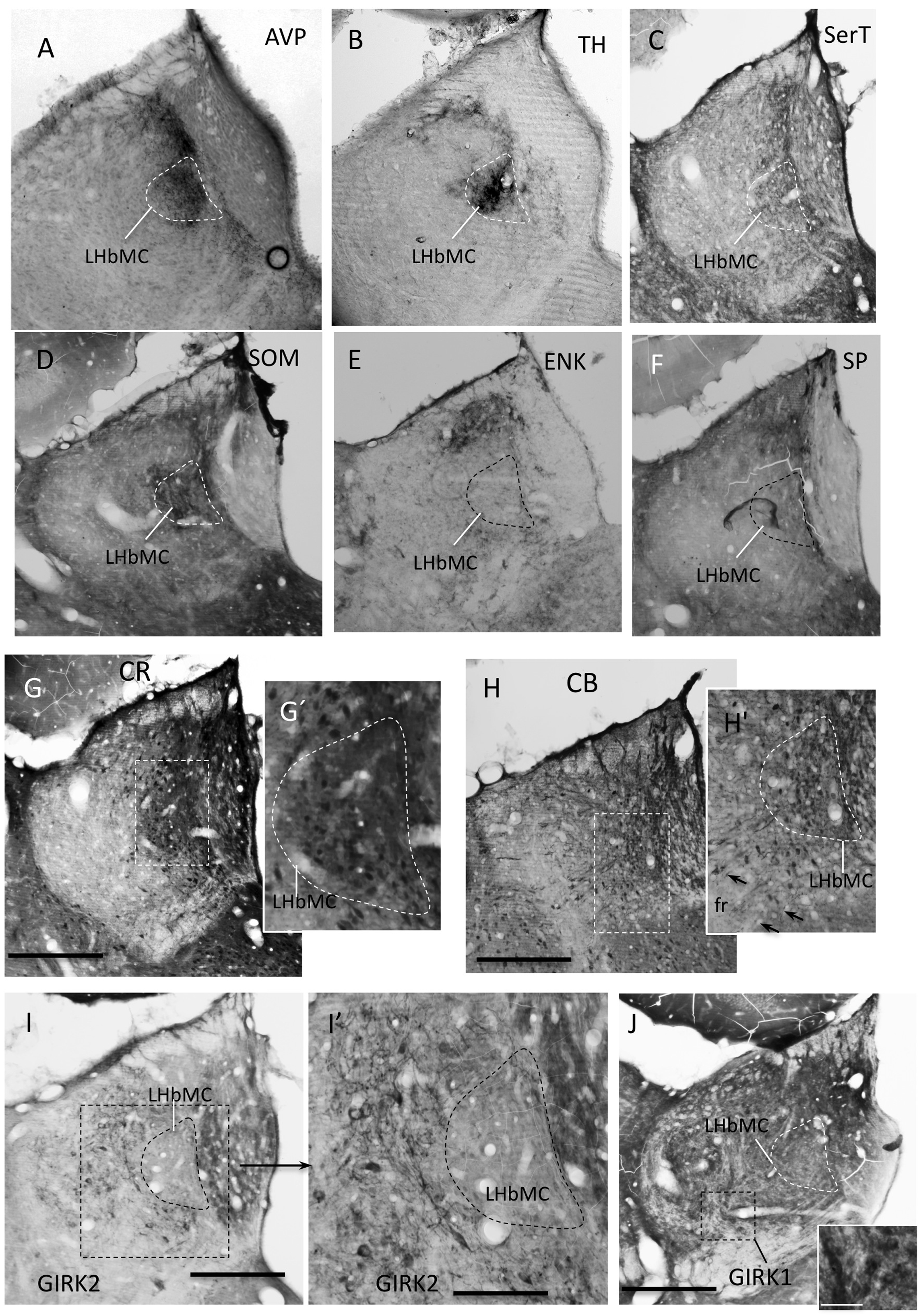

Supplement: Figure S1 — Representative photomicrographs of relevant neurochemical marker expression in habenula in relation to vasopressin innervation. Boxed regions were subsequently magnified. AVP: arginine vasopressin (A); TH: tyrosine hydroxylase (B); SerT: serotonin transporter (C); SOM: somatostatin (D); ENK: encephalin (E); SP: substance P (F); CR: calretinin (G); CB: calbindin (H); G-protein-activated inward rectifying potassium (GIRK 2) (I,I′) and G-protein-activated inward rectifying potassium (GIRK 1) (J). LHbMC: medio- central subnucleus of lateral habenula, circumscribed region. Scale bar: 100 μm. [file Image1.JPEG]

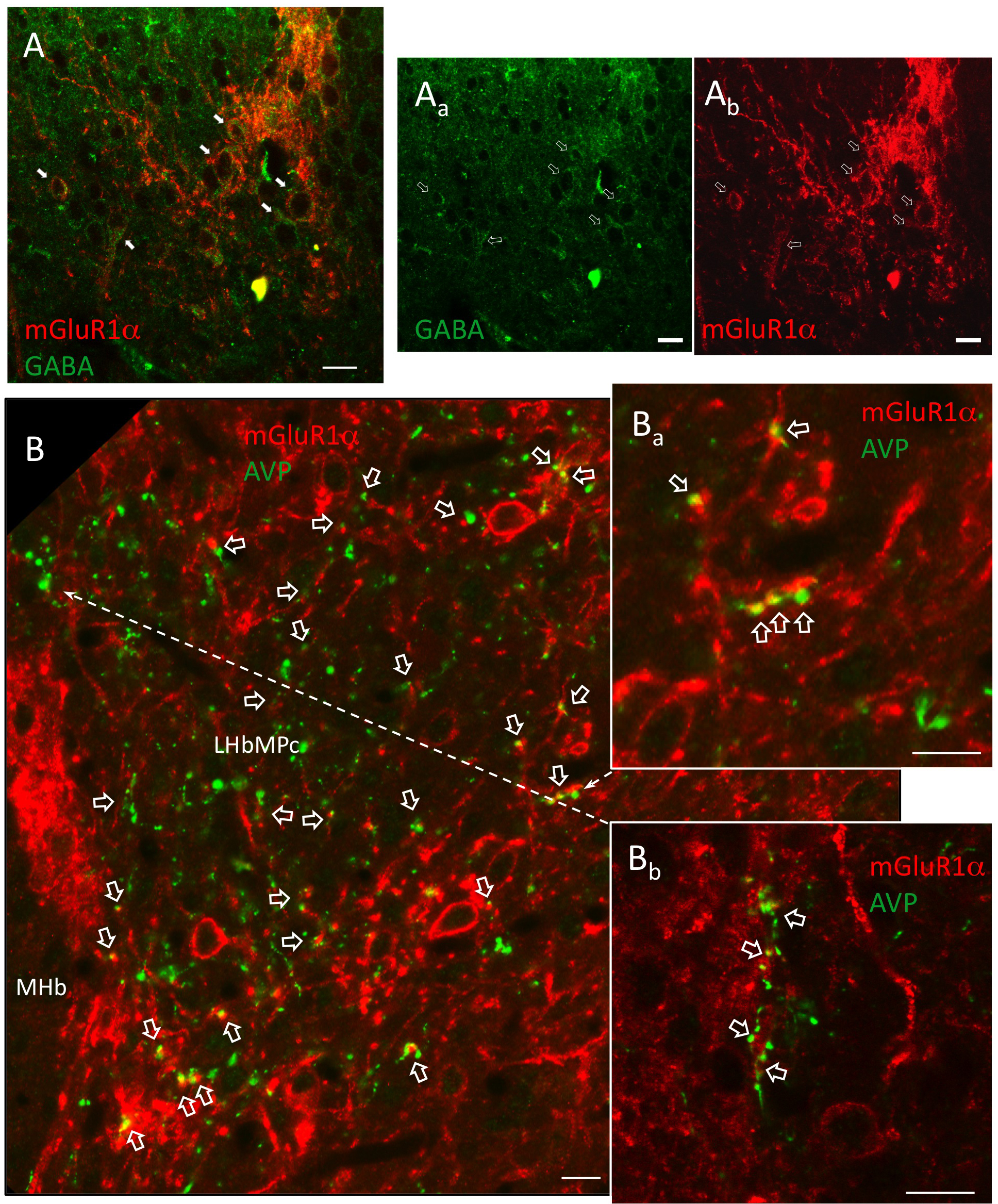

Supplement: Figure S2 — Metabotropic glutamate receptor 1α (mGluR1α) expressing GABAergic neurons were among the vasopressinergic innervation's targets. (A) Confocal images of immunohistochemical labeling for mGluR1α (red) and GABA (green), showed that numerous GABAerigic neurons, in the medial part of the lateral habenula (LHbM), had their somata and dendrites decorated by mGluR1a (arrows). (B) Confocal images of immunohistochemical labeling of AVP (green), mGluR1α (red) in LHbM. Notice that there was a strong-contacting relationship between AVP axons and mGlu1Rα expressing dendritic segments. Scale bar: 20 μm. [file Image2.JPEG]

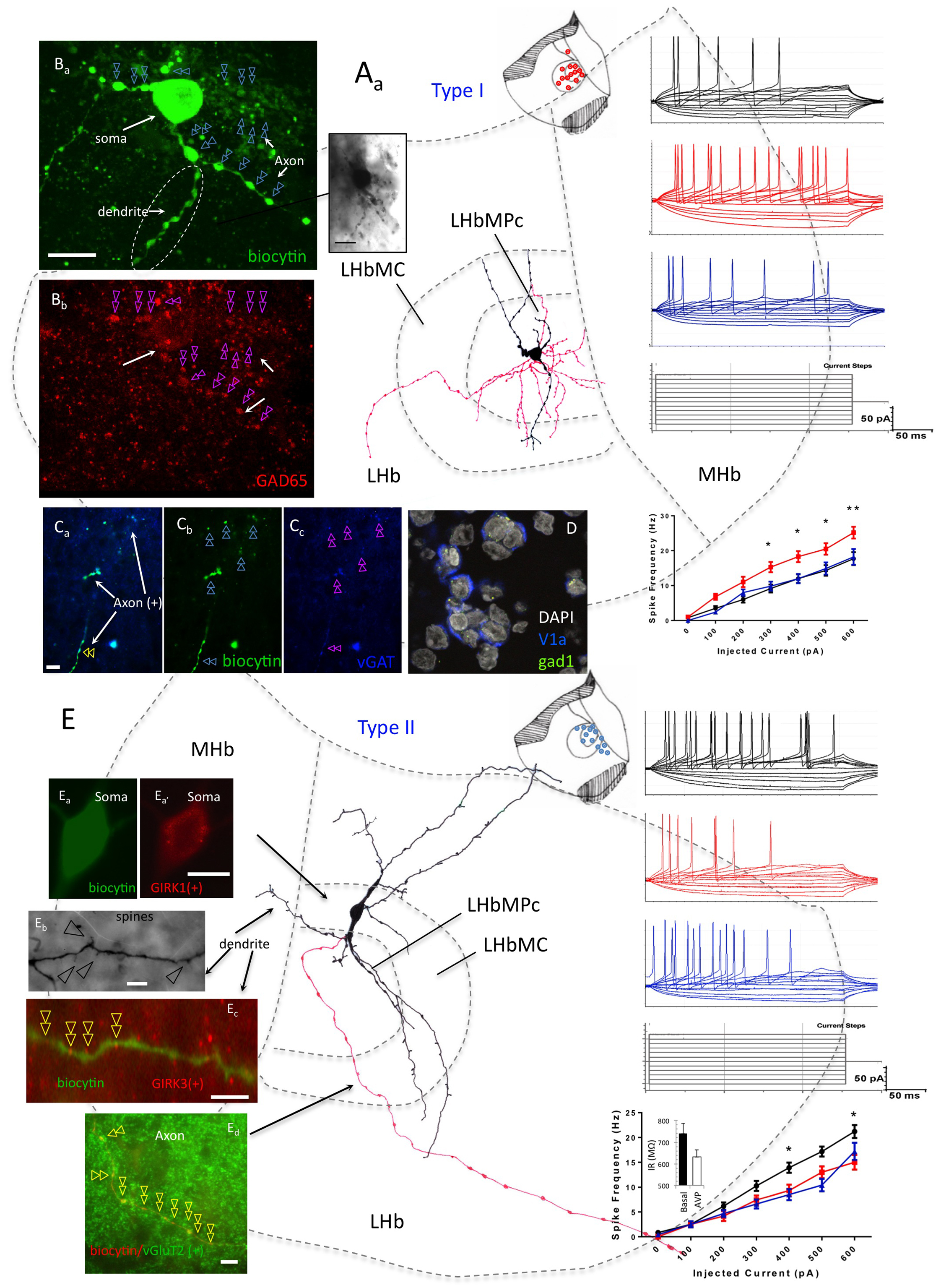

Supplement: Figure S3 — In vitro electrophysiological responses of neurons from the medio-central subnucleus of the lateral habenula (LHbMC) to 10nM AVP bath application: morphology and chemical identities. Whole-cell patch-clamp recording on the medial part of the lateral habenula (LHbM) neurons in acute coronal brain slices applying AVP (10 nM) to the recording chamber induced differential electrophysiological response: about 50% of recorded neurons increased their firing rate. Chart in panel (A) depicts the location of 11 cells, which were successfully labeled and immunoreacted as GABAergic neurons in the medio-central part of the lateral habenula (LHbMC). (Aa): a sample of reconstructed type I neuron with axons branching extensively near the soma (red lines in Aa and green biocytin labelling in Ba, indicated by double blue arrowheads), expressing GAD65 in soma and axons, and terminals [purple double arrowheads (Bb)]. The axon was detected to be VGaT+ (Cs). (D): by in situ hybridization using the RNA scope multiplex method, we found that numerous cells in this region co-express mRNAs for Gad1 (green) and vasopressin receptor type 1a (V1a, blue). Another 30% of recorded cells decreased their firing rate upon AVP application. (E): camera lucida reconstruction of a sample neuron. Its input resistance was reduced significantly upon VP bath application (histogram). Some of the cells were well-labeled with somata showing bipolar shape, with extensive dendritic arborization from both poles and a single long axon projecting to the fasciculus retroflexus. The dendrites had sparse spines (Eb). The soma and dendrite of these neurons were positive to GIRK1 (Ea and Ec). A G protein coupled inward rectified potassium channel and their axons expressed vGlut2 (Ed). Scale bars: 10 μm. [file Image3.JPEG]

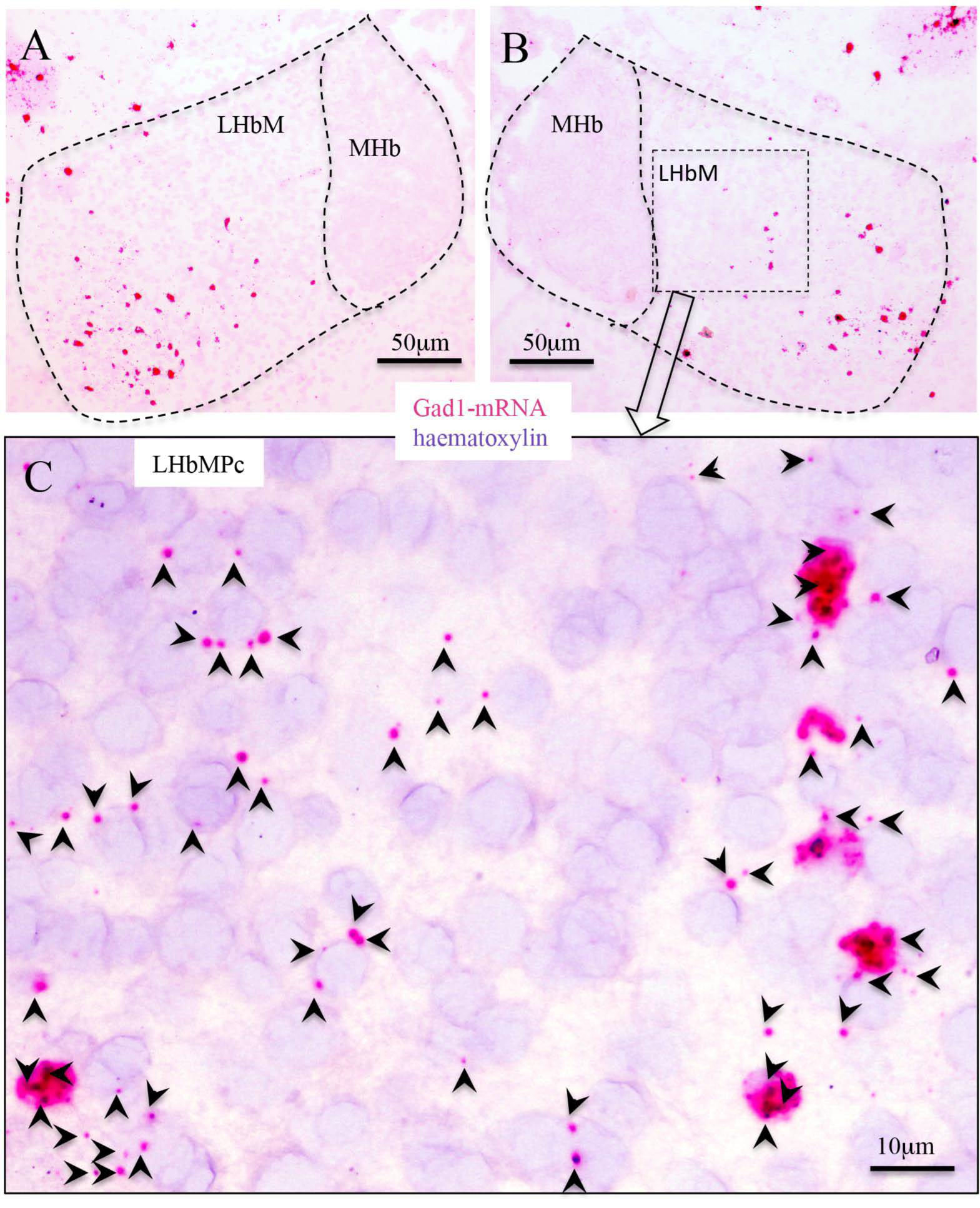

Supplement: Figure S4 — RNAscope ISH Assays confirmed the presence of Gad67 RNA in rat LHbMC. [file Image4.JPEG]
