# Supplementary material for: Thirst Is Associated with Suppression of Habenula Output and Active Stress Coping: Is there a Role for a Non-canonical Vasopressin-Glutamate Pathway?
Source: Front Neural Circuits. 2016 Mar 31;10:13. doi: 10.3389/fncir.2016.00013 (PMC4814529; doi:10.3389/fncir.2016.00013)
Supplement: Table S1 — Antibody information, related to Material and Method. [file Table2.DOCX]

| Molecule | Host species | Dilution | Source | Source code | Antibody Specificity Information |
| --- | --- | --- | --- | --- | --- |
| [Arg8 ]-vasopressin | Rabbit | 1:5000 | Peninsula-Bachem Americas, Inc., CA, USA. (www.bachem.com) | T-4563 | ([Taylor, McCarthy et al. 2008](#_ENREF_39), [Zhang and Hernandez 2013](#_ENREF_47)) |
| [Arg8 ]-vasopressin | Rabbit | 1:2000 | Prof. R.M. Buijs, Instituto de Investigaciones Biomédicas, Universidad Nacional Autónoma de México, UNAM | -- | ([Buijs, Pool et al. 1989](#_ENREF_5)) |
| Tyrosine Hydroxylase | Mouse | 1:2000 | Sigma-Aldrich Corporation, MO, USA | T-2928 | ([Li, Shi et al. 2014](#_ENREF_23), [Garcia-Aviles, Albert-Gasco et al. 2015](#_ENREF_13)) |
| Dopamine β-hydroxylase (DβH | Rabbit | 1:1000 | EMD Millipore Corporation, Billerica, MA, USA (www.millipore.com) | AB-1585 | ([Northcutt, Wang et al. 2007](#_ENREF_27), [Ragan and Lonstein 2014](#_ENREF_31)) |
| Serotonin Transporter (SerT) | Goat | 1:2000 | Santa Cruz Biotechnology, Dallas, Texas U.S.A (www.scbt.com) | SC-1458 | ([Pickel and Chan 1999](#_ENREF_29), [Ebner, Singewald et al. 2008](#_ENREF_11), [Tata and Yamamoto 2008](#_ENREF_38)) |
| Somatostatin (Som) | Mouse | 1:1000 | GeneTex, Inc., CA, USA (www.genetex.com/) | GTX7-1935, clone SOM-018 | ([Jinno and Kosaka 2000](#_ENREF_15), [Kubota, Shigematsu et al. 2011](#_ENREF_21)) |
| Enkephalin (Enk) | Mouse | 1:1000 | EMD Millipore Corporation, Billerica, MA, USA (www.millipore.com) | MAB-350 | ([Cuello, Milstein et al. 1984](#_ENREF_9), [Kenigsberg and Cuello 1987](#_ENREF_17)) |
| Substance P (SP) | Rat | 1:1000 | EMD Millipore Corporation, Billerica, MA, USA (www.millipore.com) | MAB-356 | ([Cuello, Galfre et al. 1979](#_ENREF_8), [Mai, Stephens et al. 1986](#_ENREF_24), [Diaz, Bravo et al. 2011](#_ENREF_10)) |
| Calretinin (CR) | Goat | 1:1000 | Swant Swiss antibodies, Marly, Switzerland (www.swant.com) | CG1 | ([Fuentealba, Klausberger et al. 2010](#_ENREF_12), [Unal, Joshi et al. 2015](#_ENREF_43)) |
| Calbindin | Rabbit | 1:5000 | Swant Swiss antibodies, Marly, Switzerland (www.swant.com) | CB38 | ([Sloviter 1989](#_ENREF_34), [Airaksinen, Eilers et al. 1997](#_ENREF_1)) |
| G-protein regulated Inward-Rectifier K^+^ channel, (GIRK1) | Rabbit | 1:1000 | Alomone Labs, Jerusalem, Israel (www.alomone.com) | APC-005 | ([Koyrakh, Lujan et al. 2005](#_ENREF_20), [Kim, Ma et al. 2012](#_ENREF_18)) |
| G-protein regulated Inward-Rectifier K^+^ channel, (GIRK2) | Rabbit | 1:1000 | Alomone Labs, Jerusalem, Israel (www.alomone.com) | APC-006 | ([Reyes, Fu et al. 2012](#_ENREF_32), [Booker, Gross et al. 2013](#_ENREF_3)) |
| c-Fos | Rabbit | 1:2000 | Santa Cruz Biotechnology, Dallas, Texas U.S.A (www.scbt.com) | SC-52 | ([Leao, Cruz et al. 2015](#_ENREF_22), [Soga, Teo et al. 2015](#_ENREF_36), [Velazquez, Prucca et al. 2015](#_ENREF_44), [Zuloaga, Iancu et al. 2015](#_ENREF_49)) |
| Vesicular glutamate transporter 2 | Guinea pig | 1:1000 | Frontier Institute Co., Ltd., Hokkaido , Japan (www.frontier-institute.com) | GP-AF240-1 | ([Miyazaki, Fukaya et al. 2003](#_ENREF_26), [Zhang, Qi et al. 2015](#_ENREF_48)) |
| Glutamate receptor 1C (GluR1c) | Rabbit | 1:1000 | Frontier Institute Co., Ltd., Hokkaido , Japan (www.frontier-institute.com) | GluR1C-Rb-Af692 | ([Watanabe, Fukaya et al. 1998](#_ENREF_45), [Qi, Zhang et al. 2014](#_ENREF_30), [Zhang, Qi et al. 2015](#_ENREF_48)) |
| Gamma-aminobutyric Acid (GABA) | Mouse | 1:1000 | EMD Millipore Corporation, Billerica, MA, USA (www.millipore.com) | MAB-316 | ([Kim, Ye et al. 2006](#_ENREF_19), [Tongjaroenbuangam, Jongkamonwiwat et al. 2006](#_ENREF_41), [Uematsu, Hirai et al. 2008](#_ENREF_42), [Burette, Park et al. 2014](#_ENREF_6)) |
| Gamma-aminobutyric Acid (GABA) | Mouse | 1:1000 | Sigma-Aldrich Corporation, MO, USA (www.sigmaaldrich.com) | A0310 | ([Sloviter, Ali-Akbarian et al. 2001](#_ENREF_35), [Omelchenko and Sesack 2006](#_ENREF_28), [Gonchar, Wang et al. 2007](#_ENREF_14)) |
| Glutamic acid decarboxylase 65 kDa isoform (GAD 65) | Mouse | 1:2000 | EMD Millipore Corporation, Billerica, MA, USA (www.millipore.com) | MAB351 | ([Kaufling, Veinante et al. 2009](#_ENREF_16), [Rostkowski, Teppen et al. 2009](#_ENREF_33), [Cserep, Szonyi et al. 2011](#_ENREF_7), [Stensrud, Puchades et al. 2014](#_ENREF_37)) |
| Glutamic acid decarboxylase 67 kDa isoform (GAD 67) | Mouse | 1:2000 | EMD Millipore Corporation, Billerica, MA, USA (www.millipore.com) | MAB5406 | ([Arime, Kasahara et al. 2012](#_ENREF_2), [Boulland and Chaudhry 2012](#_ENREF_4), [Margolis, Toy et al. 2012](#_ENREF_25), [Tognini, Manno et al. 2012](#_ENREF_40), [Wen, Nguyen et al. 2014](#_ENREF_46)) |

**Table SI-1: Antibody information, related to Material and Method**

**References**

Airaksinen, M. S., J. Eilers, O. Garaschuk, H. Thoenen, A. Konnerth and M. Meyer (1997). "Ataxia and altered dendritic calcium signaling in mice carrying a targeted null mutation of the calbindin D28k gene." Proc Natl Acad Sci U S A **94**(4): 1488-1493.

Arime, Y., Y. Kasahara, F. S. Hall, G. R. Uhl and I. Sora (2012). "Cortico-subcortical neuromodulation involved in the amelioration of prepulse inhibition deficits in dopamine transporter knockout mice." Neuropsychopharmacology **37**(11): 2522-2530.

Booker, S. A., A. Gross, D. Althof, R. Shigemoto, B. Bettler, M. Frotscher, M. Hearing, K. Wickman, M. Watanabe, A. Kulik and I. Vida (2013). "Differential GABAB-receptor-mediated effects in perisomatic- and dendrite-targeting parvalbumin interneurons." J Neurosci **33**(18): 7961-7974.

Boulland, J. L. and F. A. Chaudhry (2012). "Ontogenetic changes in the distribution of the vesicular GABA transporter VGAT correlate with the excitation/inhibition shift of GABA action." Neurochem Int **61**(4): 506-516.

Buijs, R., C. Pool, J. Van Heerikhuize, A. Sluiter, P. Van der Sluis, M. Ramkena, T. Van der Woude and E. Van der Beek (1989). "Antibodies to small transmitter molecules and peptides: production and application of antibodies to dopamine, serotonin, GABA, vasopressin, vasoactive intestinal peptide, neuropeptide Y, somatostatine and substance P." Biomedical research **10**(supplement 3): 213-221.

Burette, A. C., H. Park and R. J. Weinberg (2014). "Postsynaptic distribution of IRSp53 in spiny excitatory and inhibitory neurons." J Comp Neurol **522**(9): 2164-2178.

Cserep, C., A. Szonyi, J. M. Veres, B. Nemeth, E. Szabadits, J. de Vente, N. Hajos, T. F. Freund and G. Nyiri (2011). "Nitric oxide signaling modulates synaptic transmission during early postnatal development." Cereb Cortex **21**(9): 2065-2074.

Cuello, A. C., G. Galfre and C. Milstein (1979). "Detection of substance P in the central nervous system by a monoclonal antibody." Proc Natl Acad Sci U S A **76**(7): 3532-3536.

Cuello, A. C., C. Milstein, R. Couture, B. Wright, J. V. Priestley and J. Jarvis (1984). "Characterization and immunocytochemical application of monoclonal antibodies against enkephalins." J Histochem Cytochem **32**(9): 947-957.

Diaz, E., D. Bravo, X. Rojas and M. L. Concha (2011). "Morphologic and immunohistochemical organization of the human habenular complex." J Comp Neurol **519**(18): 3727-3747.

Ebner, K., G. M. Singewald, N. Whittle, F. Ferraguti and N. Singewald (2008). "Neurokinin 1 receptor antagonism promotes active stress coping via enhanced septal 5-HT transmission." Neuropsychopharmacology **33**(8): 1929-1941.

Fuentealba, P., T. Klausberger, T. Karayannis, W. Y. Suen, J. Huck, R. Tomioka, K. Rockland, M. Capogna, M. Studer, M. Morales and P. Somogyi (2010). "Expression of COUP-TFII nuclear receptor in restricted GABAergic neuronal populations in the adult rat hippocampus." J Neurosci **30**(5): 1595-1609.

Garcia-Aviles, A., H. Albert-Gasco, I. Arnal-Vicente, E. Elhajj, J. Sanjuan-Arias, A. M. Sanchez-Perez and F. Olucha-Bordonau (2015). "Acute oral administration of low doses of methylphenidate targets calretinin neurons in the rat septal area." Front Neuroanat **9**: 33.

Gonchar, Y., Q. Wang and A. Burkhalter (2007). "Multiple distinct subtypes of GABAergic neurons in mouse visual cortex identified by triple immunostaining." Front Neuroanat **1**: 3.

Jinno, S. and T. Kosaka (2000). "Colocalization of parvalbumin and somatostatin-like immunoreactivity in the mouse hippocampus: quantitative analysis with optical dissector." J Comp Neurol **428**(3): 377-388.

Kaufling, J., P. Veinante, S. A. Pawlowski, M. J. Freund-Mercier and M. Barrot (2009). "Afferents to the GABAergic tail of the ventral tegmental area in the rat." J Comp Neurol **513**(6): 597-621.

Kenigsberg, R. L. and A. C. Cuello (1987). "Role of immunology in defining transmitter-specific neurons." Immunol Rev **100**: 279-306.

Kim, S., L. Ma, K. L. Jensen, M. M. Kim, C. T. Bond, J. P. Adelman and C. R. Yu (2012). "Paradoxical contribution of SK3 and GIRK channels to the activation of mouse vomeronasal organ." Nat Neurosci **15**(9): 1236-1244.

Kim, T. J., E. A. Ye and C. J. Jeon (2006). "Distribution of AMPA glutamate receptor GluR1 subunit-immunoreactive neurons and their co-localization with calcium-binding proteins and GABA in the mouse visual cortex." Mol Cells **21**(1): 34-41.

Koyrakh, L., R. Lujan, J. Colon, C. Karschin, Y. Kurachi, A. Karschin and K. Wickman (2005). "Molecular and cellular diversity of neuronal G-protein-gated potassium channels." J Neurosci **25**(49): 11468-11478.

Kubota, Y., N. Shigematsu, F. Karube, A. Sekigawa, S. Kato, N. Yamaguchi, Y. Hirai, M. Morishima and Y. Kawaguchi (2011). "Selective coexpression of multiple chemical markers defines discrete populations of neocortical GABAergic neurons." Cereb Cortex **21**(8): 1803-1817.

Leao, R. M., F. C. Cruz, L. F. Vendruscolo, G. de Guglielmo, M. L. Logrip, C. S. Planeta, B. T. Hope, G. F. Koob and O. George (2015). "Chronic nicotine activates stress/reward-related brain regions and facilitates the transition to compulsive alcohol drinking." J Neurosci **35**(15): 6241-6253.

Li, S., Y. Shi and G. J. Kirouac (2014). "The hypothalamus and periaqueductal gray are the sources of dopamine fibers in the paraventricular nucleus of the thalamus in the rat." Front Neuroanat **8**: 136.

Mai, J. K., P. H. Stephens, A. Hopf and A. C. Cuello (1986). "Substance P in the human brain." Neuroscience **17**(3): 709-739.

Margolis, E. B., B. Toy, P. Himmels, M. Morales and H. L. Fields (2012). "Identification of rat ventral tegmental area GABAergic neurons." PLoS One **7**(7): e42365.

Miyazaki, T., M. Fukaya, H. Shimizu and M. Watanabe (2003). "Subtype switching of vesicular glutamate transporters at parallel fibre-Purkinje cell synapses in developing mouse cerebellum." Eur J Neurosci **17**(12): 2563-2572.

Northcutt, K. V., Z. Wang and J. S. Lonstein (2007). "Sex and species differences in tyrosine hydroxylase-synthesizing cells of the rodent olfactory extended amygdala." J Comp Neurol **500**(1): 103-115.

Omelchenko, N. and S. R. Sesack (2006). "Cholinergic axons in the rat ventral tegmental area synapse preferentially onto mesoaccumbens dopamine neurons." J Comp Neurol **494**(6): 863-875.

Pickel, V. M. and J. Chan (1999). "Ultrastructural localization of the serotonin transporter in limbic and motor compartments of the nucleus accumbens." J Neurosci **19**(17): 7356-7366.

Qi, J., S. Zhang, H. L. Wang, H. Wang, J. de Jesus Aceves Buendia, A. F. Hoffman, C. R. Lupica, R. P. Seal and M. Morales (2014). "A glutamatergic reward input from the dorsal raphe to ventral tegmental area dopamine neurons." Nat Commun **5**: 5390.

Ragan, C. M. and J. S. Lonstein (2014). "Differential postpartum sensitivity to the anxiety-modulating effects of offspring contact is associated with innate anxiety and brainstem levels of dopamine beta-hydroxylase in female laboratory rats." Neuroscience **256**: 433-444.

Reyes, S., Y. Fu, K. Double, L. Thompson, D. Kirik, G. Paxinos and G. M. Halliday (2012). "GIRK2 expression in dopamine neurons of the substantia nigra and ventral tegmental area." J Comp Neurol **520**(12): 2591-2607.

Rostkowski, A. B., T. L. Teppen, D. A. Peterson and J. H. Urban (2009). "Cell-specific expression of neuropeptide Y Y1 receptor immunoreactivity in the rat basolateral amygdala." J Comp Neurol **517**(2): 166-176.

Sloviter, R. S. (1989). "Calcium-binding protein (calbindin-D28k) and parvalbumin immunocytochemistry: localization in the rat hippocampus with specific reference to the selective vulnerability of hippocampal neurons to seizure activity." J Comp Neurol **280**(2): 183-196.

Sloviter, R. S., L. Ali-Akbarian, K. D. Horvath and K. A. Menkens (2001). "Substance P receptor expression by inhibitory interneurons of the rat hippocampus: enhanced detection using improved immunocytochemical methods for the preservation and colocalization of GABA and other neuronal markers." J Comp Neurol **430**(3): 283-305.

Soga, T., C. H. Teo, K. L. Cham, M. M. Idris and I. S. Parhar (2015). "Early-Life Social Isolation Impairs the Gonadotropin-Inhibitory Hormone Neuronal Activity and Serotonergic System in Male Rats." Front Endocrinol (Lausanne) **6**: 172.

Stensrud, M. J., M. Puchades and V. Gundersen (2014). "GABA is localized in dopaminergic synaptic vesicles in the rodent striatum." Brain Struct Funct **219**(6): 1901-1912.

Tata, D. A. and B. K. Yamamoto (2008). "Chronic stress enhances methamphetamine-induced extracellular glutamate and excitotoxicity in the rat striatum." Synapse **62**(5): 325-336.

Taylor, A. C., J. J. McCarthy and S. D. Stocker (2008). "Mice lacking the transient receptor vanilloid potential 1 channel display normal thirst responses and central Fos activation to hypernatremia." Am J Physiol Regul Integr Comp Physiol **294**(4): R1285-1293.

Tognini, P., I. Manno, J. Bonaccorsi, M. C. Cenni, A. Sale and L. Maffei (2012). "Environmental enrichment promotes plasticity and visual acuity recovery in adult monocular amblyopic rats." PLoS One **7**(4): e34815.

Tongjaroenbuangam, W., N. Jongkamonwiwat, P. Phansuwan-Pujito, S. O. Casalotti, A. Forge, H. Dodson and P. Govitrapong (2006). "Relationship of opioid receptors with GABAergic neurons in the rat inferior colliculus." Eur J Neurosci **24**(7): 1987-1994.

Uematsu, M., Y. Hirai, F. Karube, S. Ebihara, M. Kato, K. Abe, K. Obata, S. Yoshida, M. Hirabayashi, Y. Yanagawa and Y. Kawaguchi (2008). "Quantitative chemical composition of cortical GABAergic neurons revealed in transgenic venus-expressing rats." Cereb Cortex **18**(2): 315-330.

Unal, G., A. Joshi, T. J. Viney, V. Kis and P. Somogyi (2015). "Synaptic Targets of Medial Septal Projections in the Hippocampus and Extrahippocampal Cortices of the Mouse." J Neurosci **35**(48): 15812-15826.

Velazquez, F. N., C. G. Prucca, O. Etienne, D. S. D'Astolfo, D. C. Silvestre, F. D. Boussin and B. L. Caputto (2015). "Brain development is impaired in c-fos -/- mice." Oncotarget **6**(19): 16883-16901.

Watanabe, M., M. Fukaya, K. Sakimura, T. Manabe, M. Mishina and Y. Inoue (1998). "Selective scarcity of NMDA receptor channel subunits in the stratum lucidum (mossy fibre-recipient layer) of the mouse hippocampal CA3 subfield." Eur J Neurosci **10**(2): 478-487.

Wen, Z., H. N. Nguyen, Z. Guo, M. A. Lalli, X. Wang, Y. Su, N. S. Kim, K. J. Yoon, J. Shin, C. Zhang, G. Makri, D. Nauen, H. Yu, E. Guzman, C. H. Chiang, N. Yoritomo, K. Kaibuchi, J. Zou, K. M. Christian, L. Cheng, C. A. Ross, R. L. Margolis, G. Chen, K. S. Kosik, H. Song and G. L. Ming (2014). "Synaptic dysregulation in a human iPS cell model of mental disorders." Nature **515**(7527): 414-418.

Zhang, L. and V. S. Hernandez (2013). "Synaptic innervation to rat hippocampus by vasopressin-immuno-positive fibres from the hypothalamic supraoptic and paraventricular nuclei." Neuroscience **228**: 139-162.

Zhang, S., J. Qi, X. Li, H. L. Wang, J. P. Britt, A. F. Hoffman, A. Bonci, C. R. Lupica and M. Morales (2015). "Dopaminergic and glutamatergic microdomains in a subset of rodent mesoaccumbens axons." Nat Neurosci **18**(3): 386-392.

Zuloaga, D. G., O. D. Iancu, S. Weber, D. Etzel, T. Marzulla, B. Stewart, C. N. Allen and J. Raber (2015). "Enhanced functional connectivity involving the ventromedial hypothalamus following methamphetamine exposure." Front Neurosci **9**: 326.
